# The third international hackathon for applying insights into large-scale genomic composition to use cases in a wide range of organisms

**DOI:** 10.12688/f1000research.110194.1

**Published:** 2022-05-16

**Authors:** Kimberly Walker, Divya Kalra, Rebecca Lowdon, Guangyi Chen, David Molik, Daniela C. Soto, Fawaz Dabbaghie, Ahmad Al Khleifat, Medhat Mahmoud, Luis F Paulin, Muhammad Sohail Raza, Susanne P. Pfeifer, Daniel Paiva Agustinho, Elbay Aliyev, Pavel Avdeyev, Enrico R. Barrozo, Sairam Behera, Kimberley Billingsley, Li Chuin Chong, Deepak Choubey, Wouter De Coster, Yilei Fu, Alejandro R. Gener, Timothy Hefferon, David Morgan Henke, Wolfram Höps, Anastasia Illarionova, Michael D. Jochum, Maria Jose, Rupesh K. Kesharwani, Sree Rohit Raj Kolora, Jędrzej Kubica, Priya Lakra, Damaris Lattimer, Chia-Sin Liew, Bai-Wei Lo, Chunhsuan Lo, Anneri Lötter, Sina Majidian, Suresh Kumar Mendem, Rajarshi Mondal, Hiroko Ohmiya, Nasrin Parvin, Carolina Peralta, Chi-Lam Poon, Ramanandan Prabhakaran, Marie Saitou, Aditi Sammi, Philippe Sanio, Nicolae Sapoval, Najeeb Syed, Todd Treangen, Gaojianyong Wang, Tiancheng Xu, Jianzhi Yang, Shangzhe Zhang, Weiyu Zhou, Fritz J Sedlazeck, Ben Busby

**Affiliations:** 1 Human Genome Sequencing Center, Baylor College of Medicine, Houston, TX, 77030, USA; 2 Bayer Crop Science, Chesterfield, MO, 63017, USA; 3 Drug Bioinformatics, Helmholtz Institute for Pharmaceutical Research Saarland (HIPS), Saarbrücken, Germany; 4 Center for Bioinformatics, Saarland University, Saarbrücken, Germany; 5 Tropical Crop and Commodity Protection Research Unit, Pacific Basin Agricultural Research Center, Hilo, HI, 96720, USA; 6 Biochemistry & Molecular Medicine, Genome Center, MIND Institute, University of California, Davis, Davis, CA, 95616, USA; 7 Institute for Medical Biometry and Bioinformatics, University hospital Düsseldorf, Düsseldorf, Germany; 8 Institute of Psychiatry, Psychology & Neuroscience, King's College London, London, UK; 9 CAS Key Laboratory of Genomic and Precision Medicine, Beijing Institute of Genomics, Beijing, China; 10 Center for Evolution and Medicine, Arizona State University, Tempe, AZ, USA; 11 Department of Molecular Microbiology, Washington University in St. Louis School of Medicine, St. Louis, MO, 63110, USA; 12 Research Department, Sidra Medicine, Doha, Qatar; 13 Computational Biology Institute, The George Washington University, Washington, DC, 20052, USA; 14 Department of Obstetrics & Gynecology, Baylor College of Medicine, Houston, TX, 77030, USA; 15 Molecular Genetics Section, Laboratory of Neurogenetics, National Institute on Aging, National Institutes of Health, Bethesda, MD, USA; 16 Beykoz Institute of Life Sciences and Biotechnology, Bezmialem Vakif University, Beykoz, Istanbul, Turkey; 17 Department of Technology, Savitribai Phule Pune University, Pune, Maharashtra, India; 18 Applied and Translational Neurogenomics Group, VIB Center for Molecular Neurology, Antwerp, Belgium; 19 Applied and Translational Neurogenomics Group, Department of Biomedical Sciences, University of Antwerp, Antwerp, Belgium; 20 Department of Computer Science, Rice University, Houston, TX, USA; 21 Association of Public Health Labs, Centers for Disease Control and Prevention, Downey, CA, USA; 22 National Center for Biotechnology Information, National Library of Medicine, National Institutes of Health, Bethesda, MD, 20892, USA; 23 Department Molecular Virology and Microbiology, Baylor College of Medicine, Houston, TX, 77030, USA; 24 EMBL Heidelberg, Genome Biology Unit, Heidelberg, Germany; 25 German Center for Neurodegenerative Diseases (DZNE), Tübingen, Germany; 26 Centre for Bioinformatics, Pondicherry University, Pondicherry, India; 27 University of California Berkeley, Berkeley, CA, USA; 28 University of Warsaw, Warsaw, Poland; 29 Department of Zoology, University of Delhi, Delhi, India; 30 University of Applied Sciences Upper Austria - FH Hagenberg, Mühlkreis, Austria; 31 Center for Biotechnology, University of Nebraska-Lincoln, Lincoln, Nebraska, 68588, USA; 32 Department of Biology, University of Konstanz, Konstanz, Germany; 33 Human Genetics Laboratory, National Institute of Genetics, Japan, Mishima City, Japan; 34 Department of Biochemistry, University of Pretoria, Pretoria, South Africa; 35 Department of Computational Biology, University of Lausanne, Lausanne, Switzerland; 36 ICAR-NIVEDI, Bangalore, Karnataka, India; 37 Department of Biotechnology, The University of Burdwan, West Bengal, India; 38 Genetic Reagent Development Unit, Medical & Biological Laboratories Co., Ltd., Tokoyo, Japan; 39 Max Planck Institute for Evolutionary Biology, Plon, Germany; 40 Weill Cornell Medicine, New York, NY, USA; 41 Hoffmann-La Roche Limited, Regions, Diagnostics & Research (RDR), Mississauga, Canada; 42 Center of Integrative Genetics (CIGENE),Faculty of Biosciences, Norwegian University of Life Sciences, As, Norway; 43 School of Biochemical Engineering, Indian Institute of Technology (BHU), Varanasi, Uttar Pradesh, India; 44 University of Applied Sciences Upper Austria - FH Hagenberg, Hagenberg im Mühlkreis, Austria; 45 Max Planck Institute for Molecular Genetics, Berlin, Germany; 46 Department of Quantitative and Computational Biology,, University of Southern California, Los Angeles, CA, USA; 47 School of Biology, University of St Andrews, St Andrews, UK; 48 Department of Statistical Science, George Mason University, Fairfax, Virginia, USA; 49 DNAnexus, Mountain View, CA, USA

**Keywords:** Structural variants, k-mer, Covid-19, Long-reads, Tomatoes, Cancer, Viral integration, Hackathon, NGS

## Abstract

In October 2021, 59 scientists from 14 countries and 13 U.S. states collaborated virtually in the Third Annual Baylor College of Medicine & DNANexus Structural Variation hackathon. The goal of the hackathon was to advance research on structural variants (SVs) by prototyping and iterating on open-source software. This led to nine hackathon projects focused on diverse genomics research interests, including various SV discovery and genotyping methods, SV sequence reconstruction, and clinically relevant structural variation, including SARS-CoV-2 variants. Repositories for the projects that participated in the hackathon are available at https://github.com/collaborativebioinformatics.

## Introduction

One of the processes by which genomes incur deleterious changes are commonly linked to the genetic signatures known as structural variants (SVs). SVs are large genomic alterations, where large is typically (and somewhat arbitrarily) defined as encompassing at least 50 base pairs (bp). These genomic variants are typically classified as deletions, duplications, insertions, inversions, and translocations describing different combinations of DNA gains, losses, or rearrangements. Copy number variations (CNVs) are a particular subtype of SVs mainly represented by deletions and duplications. SVs are typically described as single events, although more complex scenarios involving combinations of SV types exist. ^1^^,^^2^ Understanding how and why SVs occur can help gain a deeper understanding of evolutionary processes driving species divergence and phenotypic adaptation, genomic processes leading to genetic variation and etiologies of plant and animal diseases. ^3^ With a recent deluge of available genomic data, SVs are an optimal target for computational biology research. ^4^

In October 2021, 59 researchers from 14 countries participated virtually in the third Baylor College of Medicine & DNAnexus hackathon, focusing on interrelated topics such as SVs, short tandem repeats (STRs), *k*-mer profiling, viruses, reference refinement and annotation. The hackathon groups addressed questions around: the use of SVs in the localization and understanding of quantitative trait loci (QTL), reference-free analysis of SVs, parallelization of SV workflows, the assessment and refining the quality of detected SVs, use of SVs in the understanding of adaptation in viruses, and understanding genetic signatures of diseases through SVs. The international hackathon focused on nine softwares to answer these questions; eight of which we present in this paper: STRdust, kTom, INSeption, GeneVar2, cov2db, K-var, Imavirus, and a Reference Panel Generator (RPG) for diverse sequencing data analysis. Several emergent themes became apparent over the course of the hackathon.

QTLs link a phenotypic trait to a local genomic region, and in its broadest definition, a molecular change affecting a phenotype. ^5^ A direct connection can be drawn between some SVs and QTLs. Linking traits and their genetic underpinnings is a common practice in the fields of agricultural genomics, molecular evolution, and genetic disease research. ^6^ Structural variation is one possible genomic change that could result in a QTL. This year’s hackathon featured work on tomatoes and other plants which provided an alternative viewpoint to the generally human-focused research of previous hackathons. Such cross-disciplinary research allows disparate groups working on similar problems to push the envelope of what is possible with current technologies.

Nucleotide sequence substrings of length *k* ( *k*-mers) continue to prove useful in SV work and in genomics, however, the time needed to assess the frequency of SVs presents a resource problem. ^7^ The reduction of the computational resources required to complete an SV assessment in a genome would allow greater amounts of SV data to be processed in genomic workflows. Many bioinformatic tools currently used to locate genomic SVs use a sliding window alignment technique, which can be time-consuming. ^8^^,^^9^ However, implementing a *k*-mer based approach to create a pool of reference *k*-mers of known SVs, the annotation speed of variation in new genomes might be increased. ^10^^,^^11^ *k*-mers have also been used in alignment-free methods, bypassing the need for reference genomes. ^12^

A portion of the hackathon focused on virus work. At the time of the hackathon, the COVID-19 pandemic was ongoing and the question of what SVs are present, and how they might change the behavior of SARS-CoV-2 was unresolved.

Together the projects of this hackathon represent a range of fields, a range of academic, industry, and government researchers, and a range of desired impacts in the field of SV analysis. Topical introductions to the specific work of each group can be found below, except from “nibSV” which was reported previously ^11^ and did not achieve significant progress.

### STRdust: Detect and genotype short tandem repeats

Short tandem repeats (STRs) ( *i.e.,* repeated instances of short 2-6 bp DNA motifs) are widespread in the genomes of most organisms. Due to their highly polymorphic nature, STRs are frequently employed in population and evolutionary genomic studies ranging from genealogy to forensics and disease diagnostics. ^13^ For example, in humans, expansions in functional STRs have been linked to many neurological and developmental disorders ^14^^,^^15^ whereas in plants, STRs have been found to impact several traits important to agriculture including growth rate and yield. ^16^ Yet, despite their importance, STRs remain relatively poorly characterized in most species. On the one hand, second-generation sequencing platforms ( *e.g.,* Illumina ^17^ (RRID: SCR_010233)) are limiting our view of STR variation within the read length due to both the short length of sequencing reads produced as well as frequent amplification biases (such as GC-biases and over−/under-representation of certain reads on a genome-wide scale). On the other hand, third-generation sequencing platforms (namely, PacBio (PacBio Sequel II System, ^18^ (RRID: SCR_017990)) and Oxford Nanopore Technologies (ONT) ^19^ (RRID: SCR_003756)) allow for the generation of single-molecule reads spanning tens to hundreds of kilobases in length but error rates (~1% in PacBio HiFi reads and ~ 10–15% in ONT ^20^) continue to exacerbate reliable STR detection. To mitigate this issue, several long-read STR calling methods have been developed in recent years, including PacmonSTR ^21^(RRID: SCR_002796), NanoSatellite, ^22^ TRiCoLOR ^23^(RRID: SCR_018801), and Straglr ^24^ – however, their usability remains limited due to platform and/or computational demands. In order to address these shortcomings, we introduce STRdust, a tool to accurately detect and genotype STRs from long reads.

### kTom: k-mers for profiling tomato introgressions

The success of commercially cultivated vegetables requires a balance of selection for domestication traits while maintaining genomic diversity and quality characteristics, and this is particularly true for tomato breeding programs. ^25^ Many desirable traits for crops are obtained by crossing elite breeding germplasm to wild relatives that carry a trait of interest ( *e.g.*, disease resistance or fruit flavor). This process of moving a genomic region from one species or distantly-related species into another is called introgression. ^25^

Tomato is an important crop and indispensable in the diet of many cultures and regions. The demand for fresh and processed tomatoes makes them one of the most important vegetables grown globally, with >180 million tons of tomatoes produced in 2019 worldwide ( FAOSTAT). ^26^

Genetic traits have been moved into cultivated tomatoes over the past several decades of tomato breeding through trait introgression. Identifying and tracking introgressed traits is a crucial function of modern tomato breeding. ^25^ The introgression of traits often occurs as large presence/absence structural variants with novel genes or sequences. Some introgressions can be completely defined by *de novo* sequencing and assembly, but this can be expensive for many samples and is not always successful for more complex genomic introgressions. ^2^ These complex structural variation patterns, coupled with the lack of reference genomes for many wild tomato relatives, complicate the efforts to locate or characterize the introgressed traits in the elite germplasm’s genome. Consequently, most marker sets today rely exclusively on SNPs, which do not always track diverse tomato genetics. ^27^

Here we present kTom, a tool to characterize the *k*-mer content of re-sequenced genomes and to identify *k*-mers that are unique to traited samples. kTom is a collection of off-the-shelf tools arranged to allow for a tractable characterization of *k*-mer frequencies in a population. We used re-sequenced tomato accessions for this demonstration, but the same approach can work for any species. Having a reference-free method to characterize and track introgression sequences will give researchers more agility to understand the nature of important traits. ^28^

### INSeption: Polishing structural variants

Some types of SVs, such as insertions, play a crucial role in shaping the genome and thus the function of each gene. For example, more than 50 percent of mammalian genomes include a repeating DNA sequence known as transposable elements. ^29^ Additionally, insertions can indicate an early tumorigenic event, ^30^ demonstrating a role in disease, making it crucial to accurately identify them.

Read-based SV calling methods broadly fall into the categories of alignment- and assembly-based approaches. ^2^ In alignment-based approaches, SVs are inferred from patterns of abnormal read mapping on an existing reference sequence. ^2^ Alignment-based approaches pose a popular method for calling SVs both from short-reads and long-reads, with a multitude of tools developed for both read mapping ( *e.g.,* BWA ^31^(RRID: SCR_010910), Minimap2 ^32^(RRID: SCR_018550), and NGMLR ^33^(RRID: SCR_017620)) and SV detection ( *e.g.,* DELLY ^34^(RRID: SCR_004603) and SNIFFLES ^33^(RRID: SCR_004603)). A downside of alignment-based SV detection lies in the incomplete resolution of complex or large genomic rearrangements or insertions exceeding common read lengths. ^35^ By contrast, assembly-based approaches utilize *de novo* sequence assemblies computed directly on the sampled reads, circumventing any biases introduced by the use of reference sequences. ^2^ SVs are thereby called by aligning such assemblies against a reference and identifying local incongruencies. Commonly used tools include Canu ^36^ (RRID: SCR_015880) and Flye ^37^ (RRID: SCR_017016) for sequence assembly, Minimap2 and BlasR ^38^ for alignment against a reference and SGVar ^35^ and Paftools ^32^ for SV calling. Assembly-based approaches can resolve even complex rearrangements and long insertions, but the construction of high-quality, haplotype-resolved assemblies requires thorough quality control and typically a high quality and diversity of data. ^39^

### GeneVar2: Gene-centric data browser for structural variants

Next-generation sequencing (NGS) technologies can be a powerful source in uncovering underlying genetic causes of diseases, but significant challenges still remain for SV interpretation and clinical analysis for clinicians. ^40^ Although various tools are available to predict the pathogenicity of a protein-changing variant—a list of these is available at OpenCRAVAT—they do not always agree, further compounding the problem. ^41^

Here we present GeneVar2: an open access, gene-centric data browser to support structural variant analysis. There are two ways to interact with GeneVar2. First, GeneVar2 takes an input of a gene name or an ID and produces a report that informs the user of all SVs overlapping the gene and any non-coding regulatory elements affecting expression of the gene. Second, users can upload variant call format (VCF) files from their analysis pipelines as input to GeneVar2. GeneVar2 will output clinically relevant information as well as provide useful visualizations of disease ontology and enrichment pathway analysis based on SV types.

### cov2db: A low frequency variant database for SARS-CoV-2

Global SARS-CoV-2 sequencing efforts have resulted in a massive genomic dataset availability to the public for a variety of analyses. However, the two most common resources are genome assemblies (deposited in GISAID ^41^ (RRID: SCR_018251) and GenBank ^42^ (RRID: SCR_002760), for example) and raw sequencing reads. Both of these limit the quantity of information, especially with respect to variants found within the SARS-CoV-2 populations. Genome assemblies only contain common variants, which is not reflective of the full genomic diversity within a given sample (even a single patient derived sample represents a viral population within the host ^43^^–^^46^). Raw sequencing reads on the other hand require further analyses in order to extract variant information, and can often be prohibitively large in size.

Thus, we propose cov2db; a database resource for collecting low frequency variant information for available SARS-CoV-2 data. As of October 2021 there were more than 1.2 million SARS-CoV-2 sequencing datasets in the Sequence Read Archive (SRA) ^47^(RRID: SCR_004891) and European Nucleotide Archive (ENA) ^48^ (RRID: SCR_006515). Our goal is to provide an easy to use query system, and contribute to a database of VCF files that contain variant calls for SARS-CoV-2 samples. We hope that such interactive databases will speed up downstream analyses and encourage collaboration.

### K-var: A “fishing” expedition for phenotype associated k-mers

*k*-mers are commonly used in bioinformatics for genome and transcriptome assembly, error correction of sequencing reads, and taxonomic classification of metagenomes. ^49^^,^^50^ More recently, *k*-mers have been used for genotyping of structural variations in large datasets in a mapping-free manner. ^51^ Sample comparison based on *k*-mers profiles provides a computationally efficient mapping-free way to address key differences between two biological conditions, avoiding the limitations of reference bias, mappability and sequencing errors. ^52^^–^^54^ Of particular interest are case-control studies, that allow to pinpoint genetic loci putatively implicated with a phenotype or a disease.

Here we develop a pipeline that takes a sample’s sequencing data from two distinct conditions (ideally control vs. treatment or two different conditions) as input and compares their *k*-mer profiles in order to highlight *k*-mers associated with the phenotype. This approach was tested in a panel of cancer cell lines from the NCI-60 dataset (RRID: SCR_003057) contrasting primary and metastatic tissues to highlight mutational signatures underlying cancer progression.

### Imavirus: Virus integration in disease

Viral infections impact human health as they can lead to short- and long-term diseases, ^55^ including cancers. Different forms of cancer are caused by viruses such as human papillomaviruses ^56^ and hepatitis B virus capable of integrating into the host genome. ^57^ Other viruses such as human immunodeficiency viruses (HIV) integrate into the host genome as a normal part of viral replication, contributing to cancer indirectly, and less commonly directly through insertional mutagenesis. ^58^ Knowing exactly where the integration events occur can help researchers and ultimately clinicians to better understand the effect of virus integration in disease.

Common assumptions about integrations are that they are single copy and show an absence of additional structural variability. ^58^ Different mechanisms might lead to different insertion site topology. For example, one would expect a difference between natural HIV-1 p31 integrase-mediated integration (insertion + tandem duplication of five bases of host target site) vs. insertion of viral genomic content (after reverse transcription in case of retroviruses like HIV) with host cell’s DNA repair machinery. Such differences might include conservation of viral terminal repeat elements with virus-specific insertion signatures ^59^ vs. divergence ^60^ from this pattern.

When considering model insertion sites for assay evaluation, insertion site location heterogeneity exists to varying degrees in natural infection (with different mechanisms such as virus-dependent integration vs. host-dependent insertion contributing differently) vs. transgenic model organism (in the case of the Tg26 HIV-1 transgenic mouse, pronuclear injection and insertion of restriction enzyme-digested pNL4–3. ^61^ NL4–3 is the most common lab strain of HIV-1. ^62^

With advances in sequencing technologies, ^63^^,^^64^ high-throughput sequencing data is available to explore viral genome integration space. Integration sites can be detected through identification of breakpoints between host and virus genome(s). ^65^ Some integrating viruses can produce run-on transcripts or may participate in trans-splicing between virus exon and downstream host exons. ^66^ Integration events have been previously detected by identifying these and other signatures such as chimeric reads in short-read sequencing (single-end and paired-end) and long-read sequencing. ^65^^,^^67^^–^^75^

Here, we suggest tools and a general workflow that can be used for virus integration detection and discuss current caveats in using publicly available datasets for this type of analysis.

### RPG: Reference Panel Generator

Despite great advances in our knowledge of NGS data analysis, a diverse complete reference genome sequence is lacking for humans. This leads to lack of sensitivity for detecting small insertions and deletions (INDELs) and structural variation, incomplete architecture of large polymorphic CNVs and correctly calling single nucleotide variants (SNVs) at complex genomic regions. High-quality Telomere-to-Telomere (T2T) CHM13 long-read genome assembly from T2T consortium ^76^ could be utilized as a reference panel to universally improve read mapping and variant calling.

Currently, we aim to provide a revised version of CHM13 reference panels along with an RPG pipeline based on 1000 Genomes Project ^77^ (RRID: SCR_006828) common allele calls and those abnormally avoided stop codons. Overall, such reference panels will greatly improve future population-scale diverse sequencing data analysis and correctly identify hundreds of thousands of novel per-sample variants in clinical settings.

## Methods

DNAnexus (RRID: SCR_011884), a cloud platform, was used to run the code developed at the hackathon. It provides flexibility to run a wide array of software applications either on a cloud workstation (default number of cores = 8) or on an interactive environment such as a Jupyter notebook (default number of cores = 16). One of these two resources were used to run the software during the hackathon, unless otherwise specified.

### STRdust

**Figure 1.**
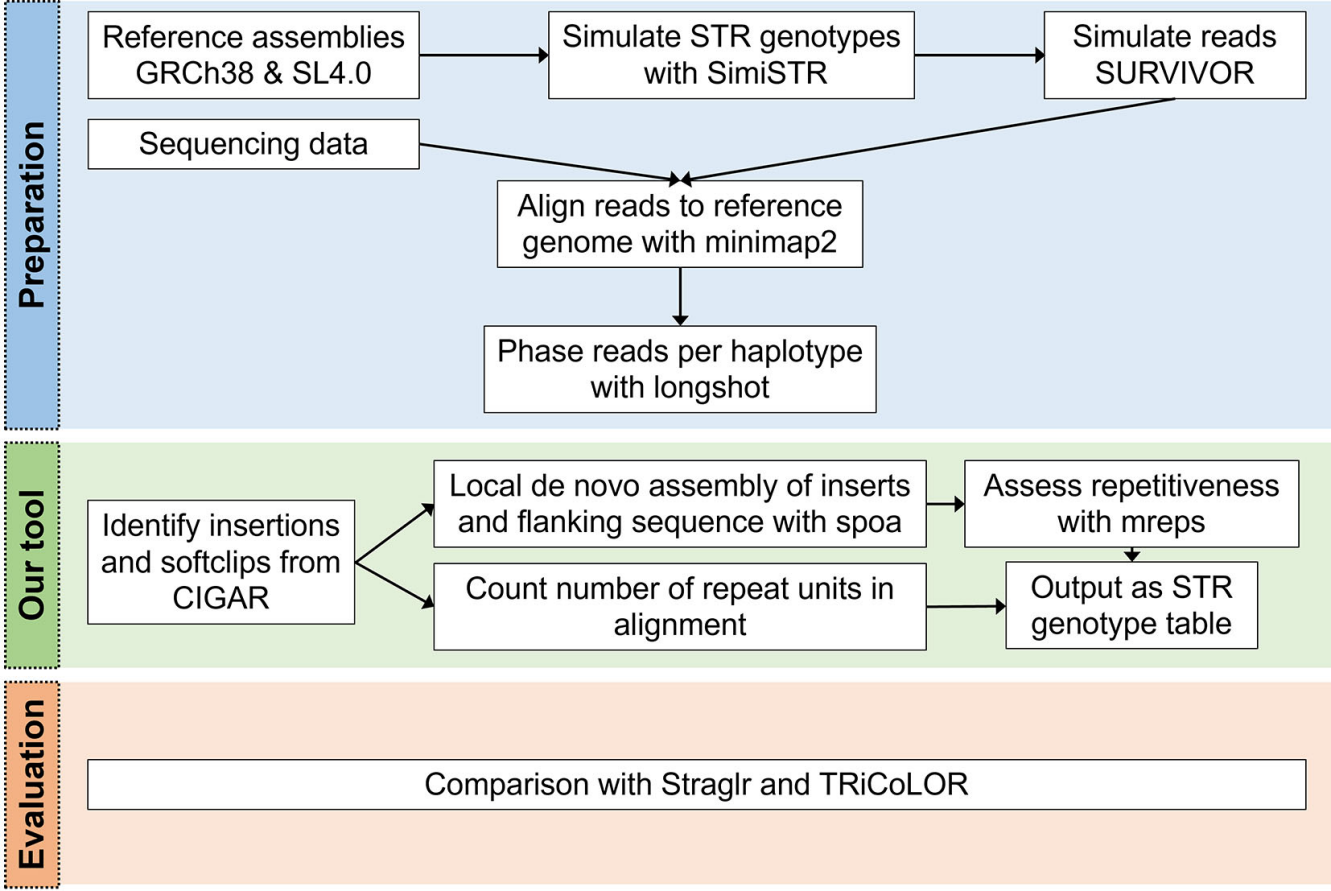
STRdust workflow. During the preparation phase, reads (either simulated or sequenced) are aligned to the corresponding reference genome with Minimap2 ^32^ and the mapped reads are then phased using longshot. Next, STRdust identifies insertions and soft-clips from the Concise Idiosyncratic Gapped Alignment Report (CIGAR) string which identify regions of possible short tandem repeats (STR) expansion. These regions are further analyzed by performing *de novo* assembly using spoa and assessing the repetitiveness of the region with mreps. STRdust outputs the STR genotype as a tab separated table for further analysis. We evaluated STRdust by comparing the results of simulated STR expansions produced by SimiSTR based on the human (Genome Reference Consortium Human Build 38, GRCh38) and tomato ( *Solanum lycopersicum* 4.0, SL4.0) reference genomes, to two novel tools: Straglr ^24^ and TRiCoLOR. ^23^

STRdust ^142^ parses the CIGAR (a compressed representation of an alignment that is used in the SAM file format) of each read, either genome-wide or in user-specified loci, in order to identify sufficiently large (>15 bp) insertions or soft-clipped bases which could indicate the presence of an enlarged STR. The sequence of those candidate-expansions is extracted, along with 50 bp of flanking sequence. Leveraging the phased input data, such insertions are combined per haplotype when multiple of these are found close by (within 50 bp) across multiple reads. The combination is done using spoa 4.0.7, ^78^ which generates a multiple sequence alignment and from that a consensus sequence. The obtained consensus sequence, in which inaccuracies inherent to the long read sequencing technologies should be reduced, is then used in mreps 6.2.01, ^79^ which will assess the repetitive character of the sequence and identify the repeat unit ( Figure 1).

STRdust was tested against simulated STR datasets produced by SimiSTR. SimiSTR modified the GRCh38 (human) and SL4.0 (tomato) reference genome assemblies. Additional variation (SNVs) was introduced with SURVIVOR 1.0.7 ^80^ at a rate of 0.001.

Long reads were simulated using SURVIVOR ^80^ for the GRCh38 (human) and SL4.0 (tomato) STR-modified genomes. Mapping was performed with Minimap2 ^32^ 2.24 two-fold (with and without the -Y parameter), and phasing was done with longshot 0.4.1. ^81^ Default parameters were used for all tools, if not otherwise mentioned. STRdust results were compared to TRiCoLOR 1.1, ^23^ and Straglr 1.1.1 ^24^ using default parameters. Figure 1 shows the workflow of STRdust described in this section.

STRdust is very easy to implement. One can, simply input the bam file after cloning the python script as follows: python3 STRdust/STRDust.py mapped_long_reads.bam -o results_dir. For further details on installation and implementation, review our github page.

### kTom

kTom ( *k*-mers for profiling Tomato introgressions) ^143^ aims to use *k*-mers to tag introgressions in elite tomato germplasm.

*Current implementation*

**Figure 2.**
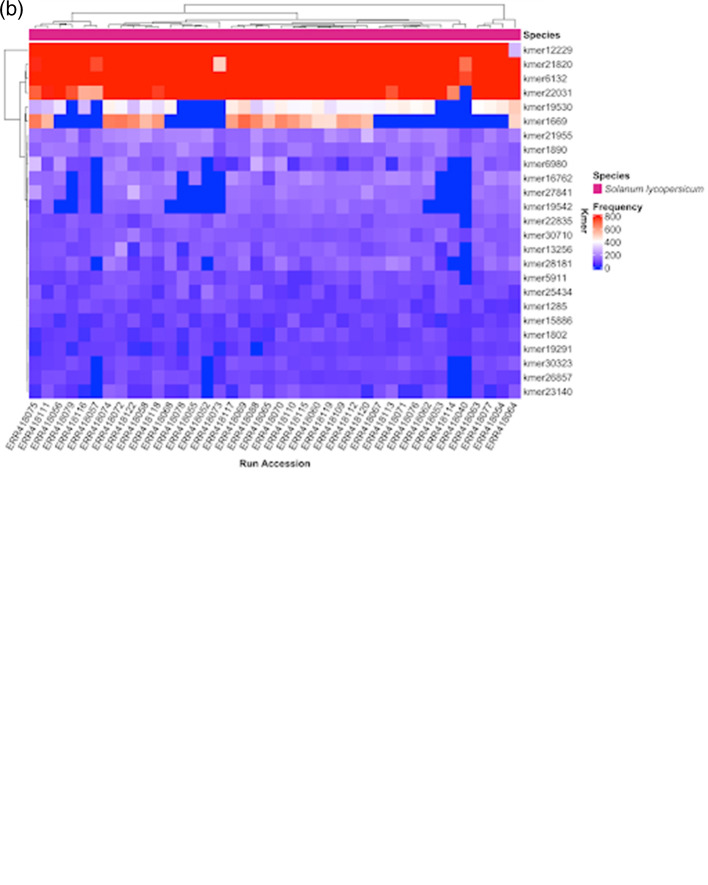
(a). kTom workflow, with major steps for individual sample and population data processing. (uniq = get unique reads; dedup = deduplicate reads). (b). *k*-mer frequency heatmap from kTom. Frequency of selected *k*-mers in each accession analyzed. Differential *k*-mer frequencies are apparent in this view. Depending on the nature of the accessions, this view may provide a first glimpse into genetic sequences underlying structural variations that differentiate the accessions.

The kTom workflow ( Figure 2a) processes re-sequenced genomes (only tested with Illumina short reads to date) to generate *k*-mer profiles per sample and calculates the population frequencies of these *k*-mers. Our use case is focused on *k*-mers with low-mid range frequencies, which we believe should capture *k*-mers unique to introgressed traits in our test population. Therefore, we use these *k*-mers to generate a distance matrix and understand the relatedness of samples.

To prototype the kTom workflow, we used 40 Whole Genome Shotgun (WGS) datasets from the 84 tomato or wild species accessions generated by The 100 Tomato Genome Sequencing Consortium ^82^ (BioProject PRJEB5235).

*Data processing*

Raw FASTQ files were quality-checked with FastQC version 0.11.9 ^83^(RRID: SCR_014583) and trimmed with Flexbar version 1.4.0 ^84^(RRID: SCR_013001), clipping five bases on 5′ and 3′ ends and keeping reads with quality score > 20 and a minimum length of 50. *k*-mers were counted using functions in Jellyfish version 2.3.0 ^85^(RRID: SCR_005491) ( jellyfish count followed by jellyfish histo) with kmersize = 21. The *k*-mers histogram was generated with Genomescope version 1.0.0 ^86^(RRID: SCR_017014). *k*-mer counts for individual samples were then aggregated into a *k*-mer frequency matrix of *k*-mers as rows and samples as columns. This frequency matrix can be visualized as an interactive heatmap (example Figure 2b) by running kmer_heatmap.R which uses ComplexHeatmap version 2.8.0 ^87^ (RRID: SCR_017270), InteractiveComplexHeatmap version 1.1.3 ^88^ and tidyverse v1.3.1 ^89^ (RRID: SCR_019186) R packages.

### INSeption

**Figure 3.**
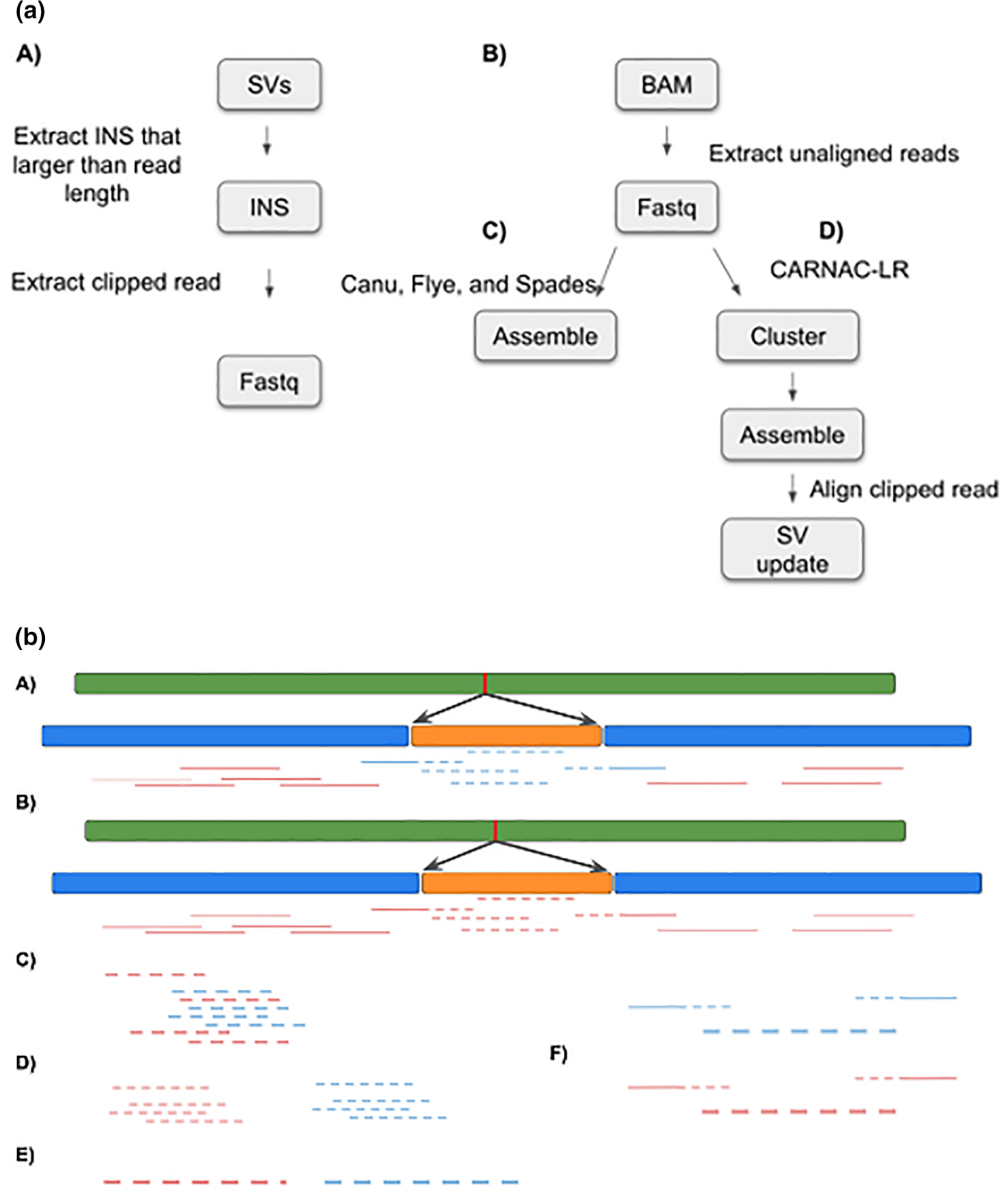
(a). INSeption workflow. Showing the tools used in the pipeline to detect insertion by extracting clipped reads (A), extracting unaligned reads (B), and then assembly (C) or clustering, assembling and aligning (D). SV: structural variant, INS:insertion, BAM: binary alignment map. (b). INSeption workflow, a graphical representation of the pipeline in (3a) showing two insertions, red and orange, in (A) and (B) we extract the unaligned reads (C), cluster them into groups (D), assemble each cluster (E) and finally align clipped reads to the assembled cluster (F).

INSeption ^144^ was tested using HiFi reads for sample HG002 (RRID:CVCL_1C78) retrieved from the genome in a bottle (GIAB) project. ^90^ The reads were aligned against GRCh37 using Minimap2 ^32^ and Sniffles 1.012 ^33^ was used to call SVs. We filtered out SVs that were supported by less than 10 reads using bcftools 1.12 ^91^ (RRID: SCR_005227). We extracted insertions that are larger than 999 nucleotides. No reads span the entire insertion. Additionally, we filtered reads that were not aligned to reference using samtools 1.14 ^91^ (RRID: SCR_002105), with the -f 4 option. Finally, we extracted reads that support each insertion studied: first, we extracted read names from the SV file using bcftools and grouped them using SV ID, followed by extracting the FASTA sequence from the binary alignment map (BAM) file using samtools and awk ( Figure 3a, left-hand side).

*Allele frequency*

For an analysis of the allele frequency (AF) for each mutation type, we created a Python ^92^^,^^93^ (RRID: SCR_008394) script (SVStat.py) that takes a VCF as input. For each SV type, it stores the AF and how often this AF was encountered. This data is then being visualized in *n* different plots (with *n* representing the number of SV types), where the x-axis represents the AF and the y-axis represents the number of times each SV type occurs.

*Clustering unmapped reads*

To be able to assemble a sequence from all unmapped reads, we tried several approaches. We attempted to identify clusters of reads using the LROD version 1.0 ^94^ package, which we found unsuitable for our purposes due to long runtimes. More successfully, we used the program CARNAC-LR version 1.0.0 ^95^ to build clusters of reads using Minimap2 version 2.22 aligner ^32^ and a subsequent *k*-mer based clustering approach. As output, for each cluster, all sequences and their IDs were exported into a FASTA file. On our testing dataset, we identified 64 such clusters. These clustered read files are then the basis for the next step for subsequent sequence assembly ( Figure 3a right-hand side).

*Delegate read clusters to the sequence assembler*

All cluster.fasta files were loaded into the assembler programs (Flye version 2.9 ^37^ and Spades version 3.15.3, ^96^ see software availability for input parameters) with another python script (clusterAssemble.py). This script has the ability to run a single cluster.fasta file or a whole batch within a directory. The inputs are the program location, program name, an optional flag: multi (for running the batch of clusters), an input directory or an input file, and an output directory ( Figure 3a right-hand side continued).

*Identifying integration sites for assembled clusters*

Having successfully assembled contigs for N = 15 read clusters using Canu v2.2 ^36^(RRID: SCR_015880), we searched for overlap of these contigs with the breakpoint regions of 30 previously identified long insertion sites. We reasoned that for each assembled contig which represents an insertion sequence, reads supporting the insertion breakpoint should also overlap with that specific contig. To find such contigs of interest, we first extracted the sequence reads (n = 604) which support a long inversion and therefore overlap at least one insertion breakpoint. This set of reads was then aligned against all 15 assembled contigs using Minimap2 (parameters: -x map-hifi -P), and using the contigs as a ‘pseudo’ reference. Finally, we manually inspected the resulting alignments to identify long (>3 kbp) contigs overlapping reads ( Figure 3b).

### GeneVar2

**Figure 4.**
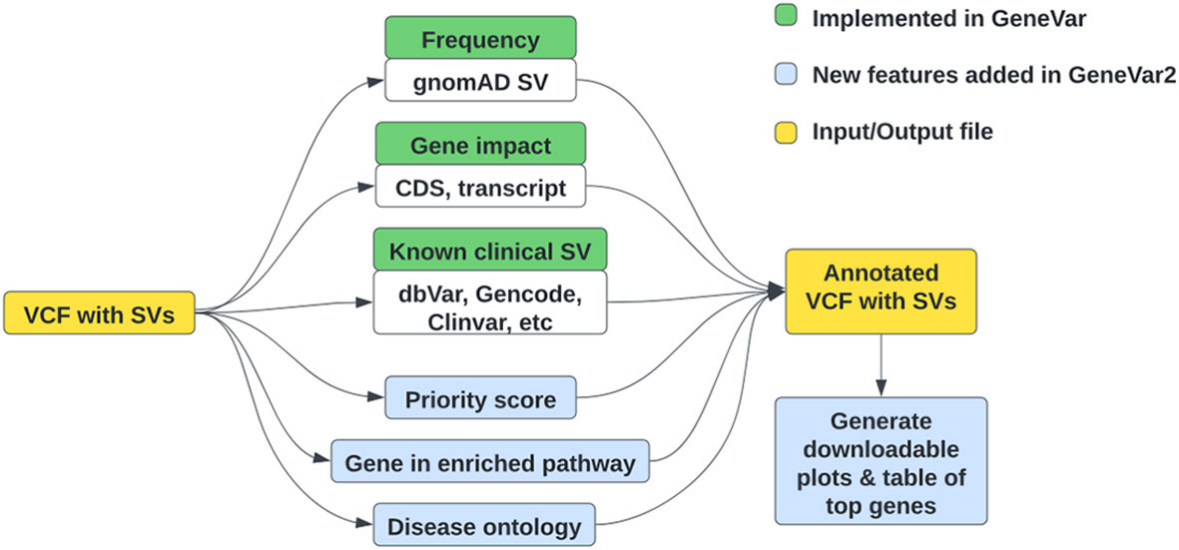
High-level outline of GeneVar2 workflow. Green boxes represent the initial features of GeneVar, implemented last year, while blue boxes represent new features implemented in GeneVar2 during this hackathon. (VCF: variant call format, SV: structural variation, CDS: coding sequence).

GeneVar2 ^145^ is an update of GeneVar, ^11^ to help inform clinical interpretation of structural variants ( Figure 4). It has expanded options allowing users to upload a VCF file, while maintaining its search functionality—based on gene name-on its web interface. GeneVar2 annotates the uploaded VCF file with a number of items which can then be downloaded by the user. Annotations include: SV allele frequency from gnomAD-SV ^85^(RRID: SCR_014964) and probability of being loss-of-function intolerant (pLI) from gnomAD; transcripts and coding regions of the impacting gene from GENCODE (v35) ^97^; the gene associations with corresponding phenotype annotation from OMIM ^100^; and known clinical SVs and their pathogenicity from dbVar. ^86^

Additionally, when a user uploads a VCF file, an option to download graphs for visualizing SVs in the dataset, is available. There is an alternate format, comma-separated values (CSV), available to download with an annotated VCF. GeneVar2, written in R, is available on GitHub (Software availability section) with detailed instructions on installation and usage. GeneVar2 is a web-based application that can also be installed by an individual on their platform to run on the command line and launch locally. Instructions on how to build and run GeneVar2 on DNAnexus can be found here.

When users launch GeneVar2 as a web-application, they can enter individual gene names (HGNC ^98^(RRID: SCR_002827)), Ensembl ^99^ (RRID: SCR_002344) gene accession (ENSG) or Ensembl transcript accession (ENST) for extracting various SVs overlapping their gene of choice. GeneVar2 will output the gene-level summary with detailed information about the SVs within the gene. It links the gene information to databases such as OMIM ^100^ (RRID: SCR_006437), GTEx ^101^(RRID: SCR_013042), gnomAD and allele frequency is reported based on gnomAD genomes and exomes.

If users first need to call SVs on their samples, the developers recommend Parliament2 ^102^(RRID: SCR_019187). Parliament2 runs a combination of tools to generate structural variant calls on whole-genome sequencing data. It can run the following callers: Breakdancer ^103^(RRID: SCR_001799), Breakseq2, ^104^ CNVnator ^105^(RRID: SCR_010821), Delly2, ^34^ Manta, ^106^ and Lumpy ^107^(RRID: SCR_003253). Because of synergies in how the programs use computational resources, these are all run in parallel. Parliament2 will produce the outputs of each of the tools for subsequent investigation. See the Parliament2 GitHub page for further details.

After users upload a VCF file containing SVs, GeneVar2 annotated each entry with the genes overlapping the SV, allele frequency from gnomAD-SV, and assigns a clinical rank to all the SVs in the VCF relative to each other. This is accomplished using the main annotation script *annotate_vcf.R.* The final annotated file is available for download as a VCF and CSV format. For Gene and Disease ontology and pathway analysis, *GeneAnnotationFromCSV. R* supports the enrichment analysis using KEGG ^108^^–^^110^(RRID: SCR_012773), Disease Ontology (DO), ^111^ Network of Cancer Gene ^112^ and Disease Gene Network (DisGeNET) ^113^ (RRID: SCR_006178). In addition, several visualization methods were provided by Bioconductor package *clusterprofiler* ^114^ (RRID: SCR_016884) and *enrichplot* ^115^ to help interpreting enrichment and disease ontology results.

Alternatively, if users prefer they can run GeneVar2 on the command line, by installing it on their platform. Users should have R version 4.1 or higher installed. In addition, you will need to have *sveval,* a custom R library, installed which can be accessed via *BiocManager* using *‘jmonlong/sveval’.* Scripts and instructions can be found on GeneVar2’s Github repository in the software availability section.

### cov2db

**Figure 5.**
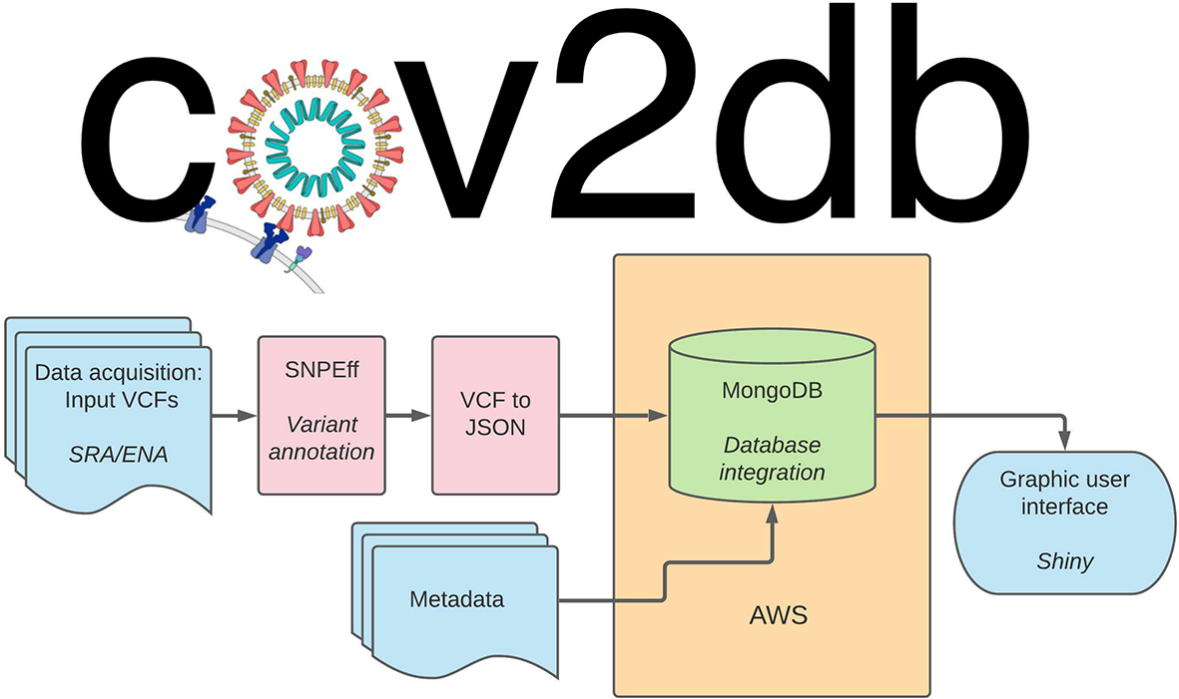
Cov2db workflow architecture. User provided variant call format (VCF) (or iVar output) files are annotated and ultimately converted into JavaScript object notation (JSON). The resulting JSON files serve as the primary input into the database. Secondary input can be provided by supplying any relevant metadata with the sample accession numbers serving as key. The resulting database can be queried directly via mongoDB command-line interface (CLI) or summarized and presented visually via the corresponding R Shiny app. AWS: Amazon web services.

cov2db ^146^ is implemented as a set of modular scripts which enable the user to annotate and reformat their original VCF files into mongoDB (RRID: SCR_021224) ready JavaScript object notation (JSON) documents. Namely, there are three key components provided within the code repository ^1^: the VCF annotation and processing framework, together with the relevant software and scripts ^2^; a sample set of annotated VCFs that can be used as a starting point for a SARS-CoV-2 iSNV database ^3^; an R Shiny ^116^ (RRID: SCR_001626) app to facilitate a graphical user interface (GUI) for the interactions and quick summaries of the data within the database ( Figure 5). The fields to query the cov2db database, such as annotation and variant information, are listed in the readme on our Github page. All of the above can be used to spin up an independent instance of cov2db and provide a user interface to interact with it. Minimal system requirements for a local cov2db instance are dictated by the mongoDB requirements with the key limiting factor being RAM used. Large variant databases will consume substantial amounts of RAM, and we suggest hosting those on dedicated high memory compute servers. Cov2db can run on x86 *nix-style platforms as is. We have not tested the software on ARM architectures or Windows based hosts. End users can interact with a hosted database from any web browser.

Our current design supports input VCFs generated by LoFreq ^117^ (RRID: SCR_013054) or converted into VCFs from the iVar ^118^ output via provided script. These files are subsequently annotated with snpEff ^119^ (RRID: SCR_005191) using the SARS-CoV-2 reference, and resulting information is recorded as an annotated VCF. Finally, we provide an additional script to convert the annotated VCFs into JSON files that can be directly integrated into the mongoDB database. Metadata intake for the database is separate, and linking between the metadata for the samples and the variant call data is done within the database via the accession number keys.

### K-var

**Figure 6.**
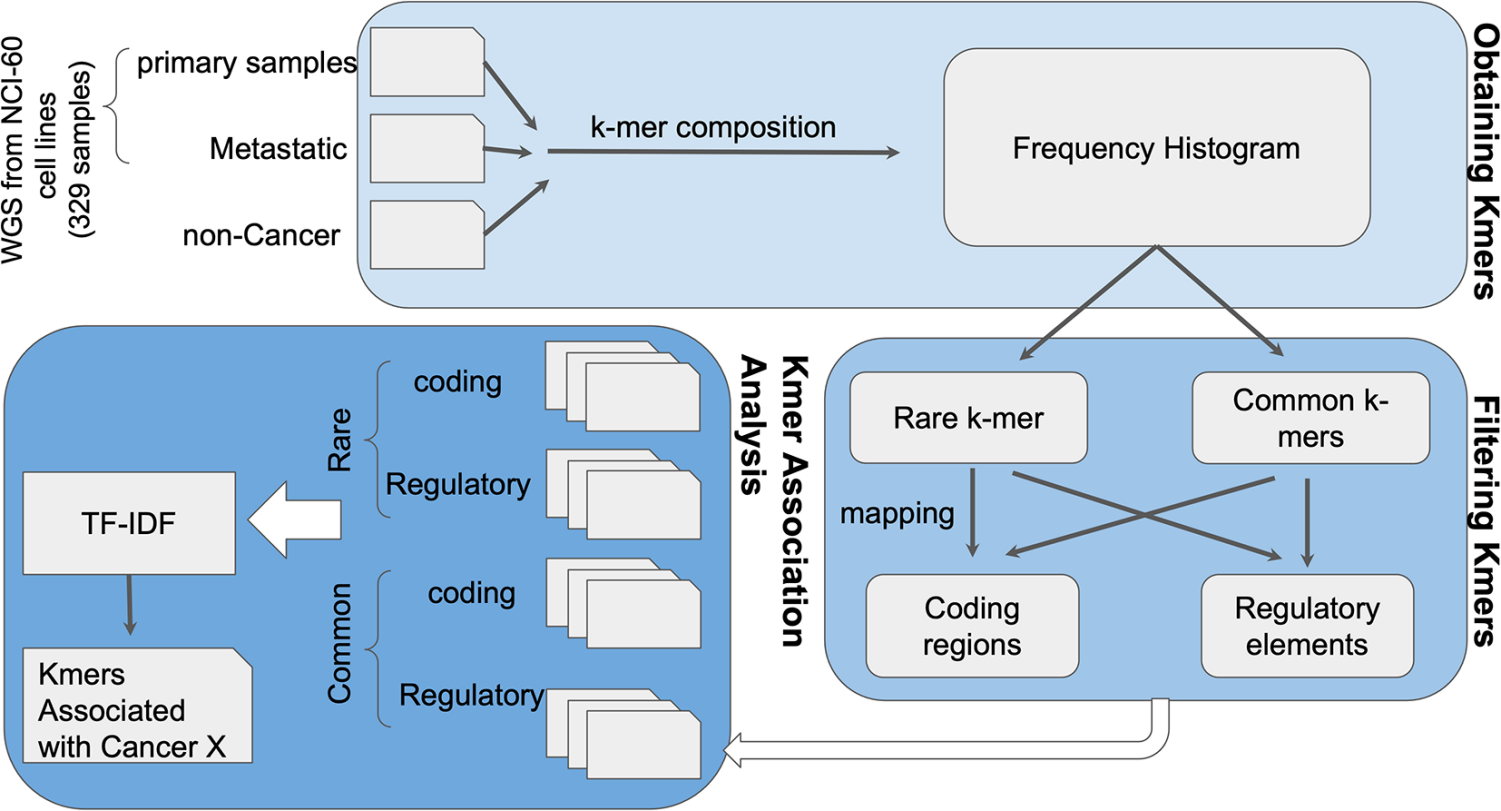
K-var workflow. The *k*-mer composition of whole-genome sequencing (WGS) sequencing data from cases and controls is obtained using Jellyfish. Rare and common *k*-mers are identified based on their frequency across samples, and mapped to a reference genome to assess their putative functional impact. Selected *k-*mers are then compared between cases and controls using term frequency-inverse document frequency (TF-IDF) statistical modeling to evaluate association with the phenotype of interest. As a proof of concept, K-var was implemented using cancer samples from the NCI-60 dataset.

As a proof of concept for K-var, ^147^ we used whole exome sequencing of the NCI-60 dataset, a panel of 60 different human tumor cell lines widely used for the screening of compounds to detect potential anticancer activity ( Figure 6). *k*-mer frequencies were obtained for each sample, using the tool Jellyfish version 2.3.0. First, counts of *k*-mers of size 31 were obtained with jellyfish count. Using a custom script, *k*-mers sequence and counts were tabulated to facilitate downstream analyses. The frequency distribution was plotted using R v3.6.3 ^120^ (RRID: SCR_000432), and low frequency *k*-mers likely arising from sequencing errors were removed. We measured the relevance of *k*-mers to the condition using TF-IDF (term frequency-inverse document frequency) with pre-defined control and test datasets. *k*-mers significantly correlated to the disease are extracted using logistic regression followed by ranking and/or classification of the significant *k*-mers. The genomic positions of the disease associated *k*-mers were identified and these positions were run through the ensembl-VEP pipeline to detect probable biological consequences.

### Imavirus

There’s an abundance of public high-throughput sequencing data (e.g. via the National Center for Biotechnology Information Sequence Read Archive). Some integrating viruses can produce run-on transcripts or may participate in trans-splicing between virus exon and downstream host exons. ^121^ Others have shown that it is possible to identify integration events by identifying chimeric reads in single-end short-read and paired-end short-read sequencing, as well as long read sequencing. ^65^^,^^67^^–^^75^ Others have not yet interrogated available large public datasets with current iterations of mapping. ^148^

**Figure 7.**
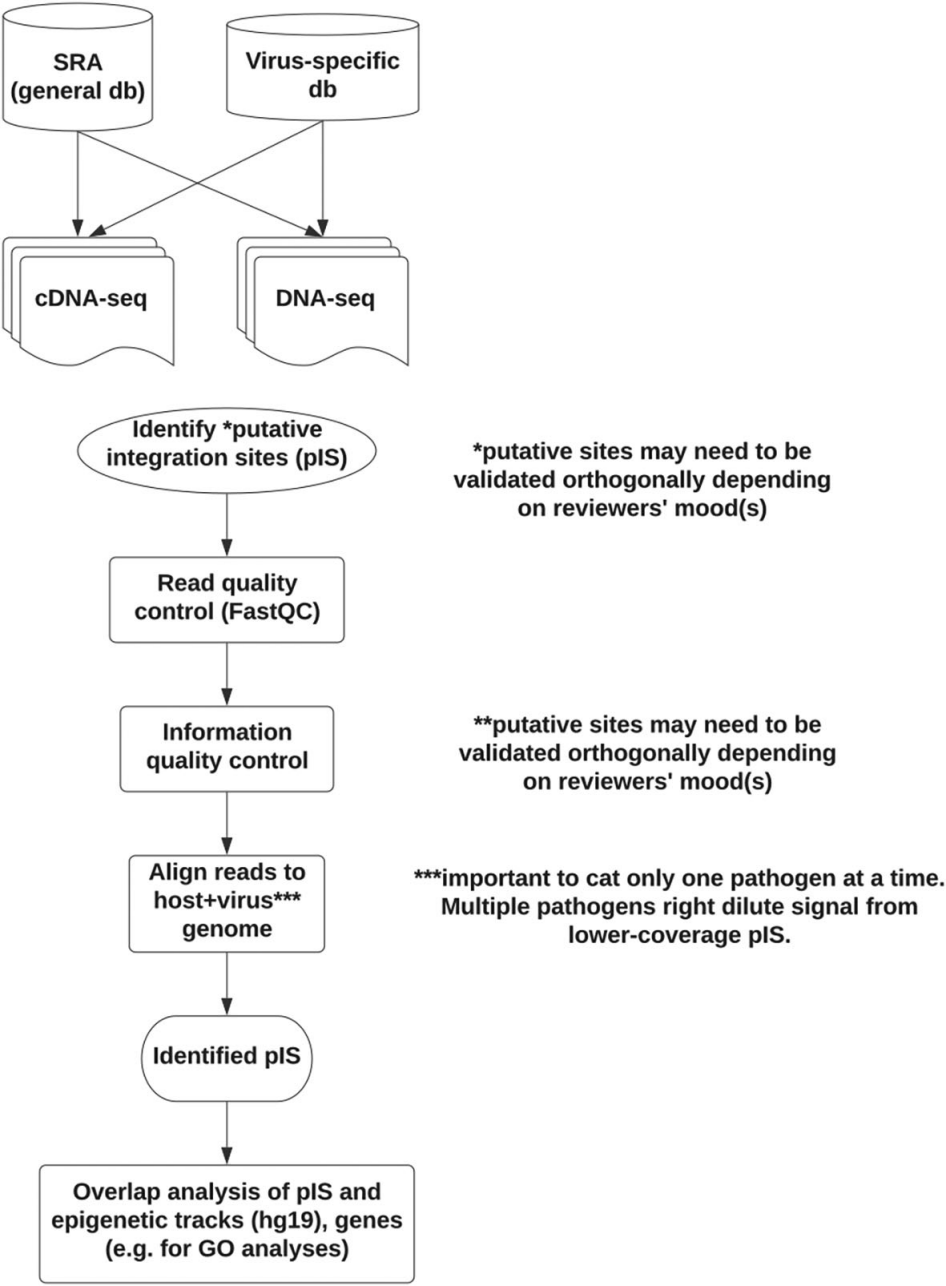
Imavirus workflow. To scope out the samples relevant for viral integration studies, human viruses known to integrate were chosen, along with viruses believed not to integrate (negative control set). Not shown, a dataset to contain human immunodeficiency virus (HIV) sequence (Tg26) and to express HIV protein was used as a positive control for pipeline development. Sequence Read Archive (SRA) was evaluated for the presence of RNA-seq (expression) and DNA-seq (host genomic DNA) from relevant viruses. A generic pipeline was evaluated on the positive control dataset with the goal of processing viral samples in SRA. Future work would also evaluate identified insertion/integration sites for possible clinical relevance. (GO: Gene Ontology).

We sought to do so by scoping out the available data and exploring at least one control dataset. We then generated a non-exhaustive list of relevant human pathogenic viruses and evaluated tools for unbiased interrogation of paired-end short-read data. Minimap2 version 2.22, ^32^ HISAT2 version 2.2.1 ^122^ (RRID: SCR_015530), and STAR version 2.7.9a ^123^ (RRID: SCR_004463) were evaluated on paired-end short-read RNA-seq from the Tg26 mouse model with HIV believed to be inserted as a transgene. Minimap2 did not work for visual exploration by default, possibly because it treats paired-end reads as single-end. Mapped reads were viewed in IGV colored by orientation and with “view as pairs” selected. HISAT2 and STAR, both split-read mappers, worked to identify at least one previously identified insertion site on mouse chr8. ^124^ Finally, we refined this approach using human plus individual virus genomes ( Figure 7).

The mouse model used includes two “insertion sites” on chr8, one on chr18, two on chrX, and a camouflaged one on chr4 embedded in a LINE element (the last site validated by long-read sequencing and deep paired-end 150 genomic DNA sequencing). These sites segregated together when multiple animals were genotyped and sequenced. ^125^^,^^126^ This behavior is suggestive of a yet to be defined complex structural variation encompassing multiple HIV transgene “copies” together with parts of different mouse chromosomes. The Tg26 HIV-1 transgenic mouse model ^61^ illustrates the current limitations of using short-read sequencing, which may only capture virus:host junctions (insertion/integration half-sites) in the absence of recapitulating the entire insertion site unambiguously. When deriving putative viral integration sites from RNA-seq, sites may be more likely to be detected if coming from highly expressed loci.

### RPG

**Figure 8.**
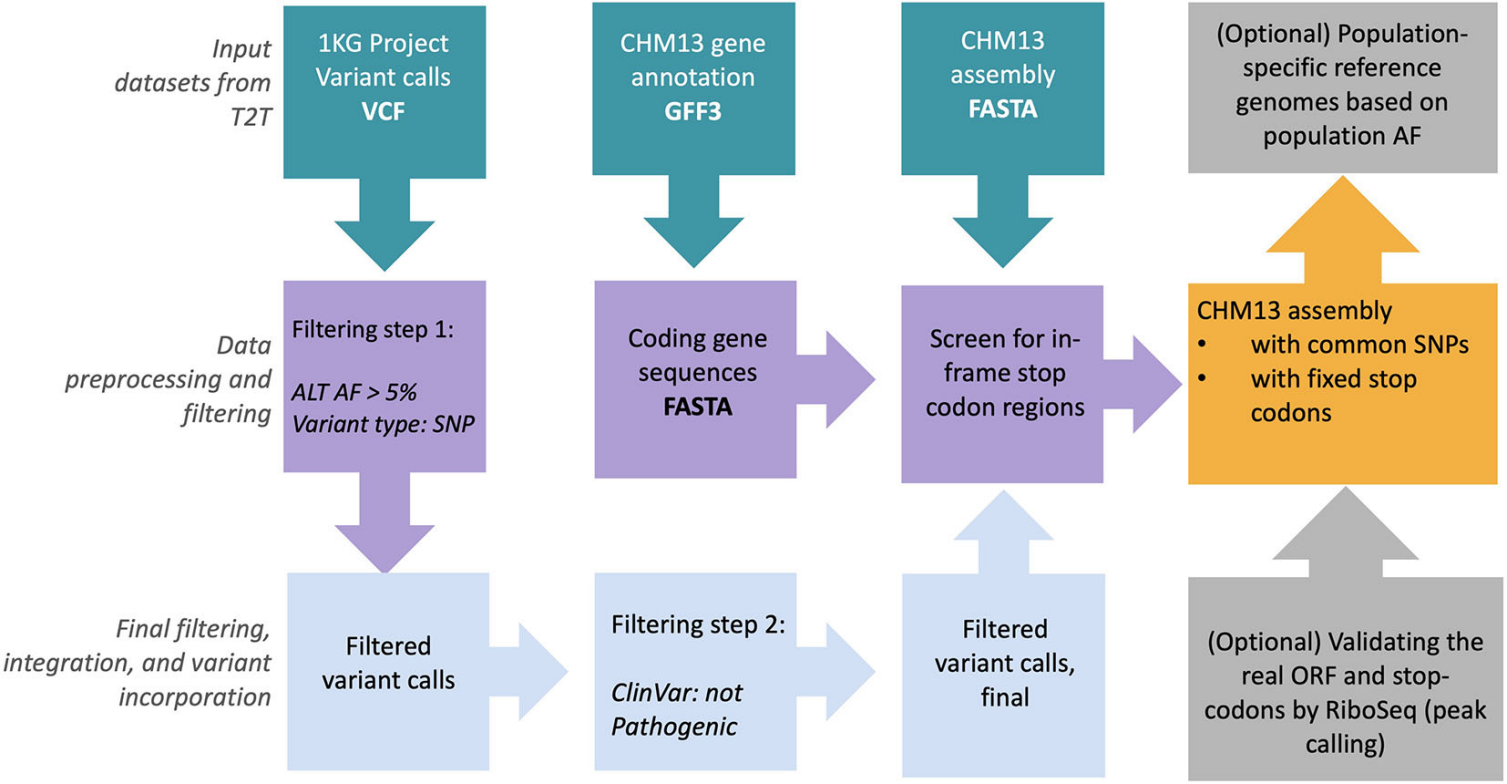
Overview of the reference panel generator pipeline for revising CHM13 reference panel. CHM13 genome sequence (FASTA), gene annotations (GFF3), and combined 1000 Genomes Project single nucleotide variants (SNVs) and insertion/deletion (INDEL) call sets in variant call format (VCF) are retrieved from Amazon-AWS ^127^ (RRID: SCR_012854) cloud. Only common alleles (>5% allele frequency (AF)) in the variant call set are retained. ClinVar ^128^ database was used to annotate variant calls with any clinical significance. Subsequently, common allele calls are replaced with CHM13 rare alleles in CHM13 FASTA genome sequence. Finally, screen-out in-frame stop-codon sites from genome sequence in order to generate the final reference panel files in FASTA format.

RPG ^149^ is a scalable and easy to apply pipeline that utilizes input genome assembly (FASTA format) and gene annotations (GFF3 format), and outputs reference panels based on the 1000 Genomes Project (1KGP) common allele calls and those abnormally avoided stop codons. Currently, the RPG pipeline is tested on the T2T-CHM13 genomic data set provided by T2T consortium in an effort to provide high-quality reference panels for diverse sequencing data analysis ( Figure 8). The generation of this panel is described in Figure 8 and the accompanying figure legend.

The resultant output T2T genome features completeness (i.e. filled gaps in its genomic sequence) compared to previously available GRCh38 releases. It further harbors 1KGP common alleles and avoids stop codons. Such T2T genomic sequence can be utilized in the ‘read mapping’ and ‘variant calling’ steps while processing whole genome sequencing (WGS) data and has important applications in improving structural variant identification. The output files generated by RPG pipeline are available in GitHub repository (Software availability section) along with supplementary pre-processing scripts.

## Use cases

Please refer to the Methods section for implementation details of the software including its input/output options and dependencies.

### STRdust

Identification and characterization of STR using short-read sequencing data have been met with shortcomings including biases introduced by polymerase chain reaction (PCR) amplification. Long-read sequencing can identify STRs more accurately than short-read sequencing as reads can span across the entire repeat region, however, they still exhibit a high error rate. Although tools have been developed to address this problem, they still have limitations such as not being able to consider multiple STRs in a single read. To address this, our tool STRdust is capable of detecting and genotyping STRs in long-read sequencing data in both mammals and plants without prior genome annotation. As a proof of concept we simulated STRs expansions using the human and tomato reference genomes and current annotations and applied STRdust, which only requires a long read sequence alignment (see Methods). This tool can be used by plant breeders to accurately genotype STRs and develop linkage maps, which are essential for mapping quantitative trait loci and intelligently selecting for combinations of traits of interest in the offspring.

### kTom

In plant breeding, important traits are often moved into elite breeding material through traditional plant breeding methods of crossing and back-crossing with phenotypic selection to retain the trait of interest. In the era of genomics, genotype markers can be used to track the introgression of traits into different lines. However, for traits with complex underlying genome biology, including structural variations, SNP-based markers are often insufficient to discover or track traits reliably in a breeding pipeline. This is particularly relevant for identifying and tracking disease resistance loci, which have been introgressed from wild tomato relatives into elite tomatoes over decades of breeding ^25^ and higher-resolution tracking of those loci could accelerate tomato breeding. To circumvent the SNP-based limitations for finding and following trait introgressions, the kTom tool uses a *k*-mer approach to characterize re-sequenced genomes and identify potential *k*-mer tags for trait introgressions. The kTom tool enables the user to understand the *k*-mer profile of the resequenced genome (from Illumina WGS reads) and compare that to a background *k*-mer profile (e.g. the reference genome for that line or a known genome without the trait of interest) to identify novel *k*-mers. kTom can enable population-level analysis of structural variation, including establishing an alternate (non-SNP) genotyping method to profile introgressions within a population and investigating and visualizing the history of introgressions. A derivation of kTom data can facilitate understanding tomato population structure with a data type more able to account for SVs. In addition, the output of kTom should be able to form the basis for a *k*-mer GWAS approach. ^28^ The kTom tool was designed with plant breeding problems in mind, but it can be applied to any resequencing dataset without the need for a reference genome.

### INSeption

Insertions play an important role in human genetic variability and diseases, and therefore their accurate identification is key for genetic analyses and clinical studies. However, comprehensively identifying sequence-resolved insertions can be challenging, especially when the read length is not sufficient to span the whole inserted sequence. In those cases, SV callers will identify the insertion’s location but not its sequence. INSeption is a bioinformatics workflow that addresses this issue by reconstructing the inserted sequence utilizing the unaligned portions of reads (i.e. hanging reads). After retrieving a sample’s unaligned reads, INSeption builds a consensus sequence to provide sequence-resolved insertions. This information allows scientists to better assess the impact of an insertion on gene function and genome organization.

### GeneVar2

SVs account for more genetic differences between humans than other types of variation and are the underlying genetic cause of several traits and diseases. ^33^^,^^129^ Although SV discovery has become more readily available, its interpretation is particularly challenging for those outside the immediate field of genetics. ^40^^,^^41^ GeneVar2 is an extremely fast and computationally efficient platform for the analysis, visualization, and interpretation of structural variation data. It is designed to provide a powerful and easy-to-use tool for applications in biomedical research and diagnostic medicine at minimal computational cost. Its comprehensive approach brings the analyses of structural variation within the reach of non-specialist laboratories and to centers with limited computational resources available.

### cov2db

Cataloging viral mutations within a sample (intra-host variation) and across samples (inter-host variation) provides critical insights to understanding the dynamics of viral evolution during the COVID-19 pandemic. ^130^ The SARS-CoV-2 virus has been shown to have high genomic diversity ^45^^,^^131^), and mutations can change the fitness of the virus ^132^ by increasing its transmission or pathogenicity potential. ^133^^,^^134^ SNVs can also result in dramatically different protein function and recognition, ^135^^,^^136^ and studies have shown persistent intra-host evolution of SARS-CoV-2 in immunocompromised hosts. ^137^ cov2db represents an integrative platform and complementary database for active monitoring of SARS-CoV-2 strain variants specific to circulating SARS-CoV-2 lineages and will facilitate efficient and sensitive tracking of both inter-host and intra-host SARS-CoV-2 variation.

Input to cov2db consists of a single or multiple VCF file(s) in the format output by the LoFreq variant caller. Cov2db does not provide an output, but allows its users to interact with a mongoDB database instance containing the variant calling information provided by the users.

### K-var

The identification of phenotype-associated biomarkers is crucial for precision medicine, crop breeding, and answering evolutionary questions. Based on the area of interest, K-var can be applied to identify mutational signatures that help distinguish between conditions using phenotype associations with low bias. Short-read sequencing data is used as input to estimate *k*-mer frequencies per sample, followed by statistical correlation to a known phenotype across two distinct conditions. The output is a ranked table of significant phenotype-associated *k*-mers that can be used to fish for genomic regions experiencing mutations. Precisely identifying these genomic locations will help in downstream analysis to infer biological consequences. During the hackathon, we ran K-var using as input metastatic and non-metastatic breast cancer whole-exome sequencing (non-metastatic *n* = 7; metastatic *n* = 5) from the NCI-60 dataset. K-var delivered a ranked list of 44,884 *k*-mers, where the score indicates the relevance of each sequence (calculated by TF-IDF analysis) in differentiating metastatic and non-metastatic (primary) sequences. The top-ranked *k*-mer identified by K-var impacted methyl-methanesulfonate sensitivity 19 ( *MMS19*), a component of the Fe-S assembly machinery involved in the production of proteins associated with genomic stability, such as DNA polymerase and DNA repair proteins. This gene has been reported as a breast cancer candidate gene in familial studies in Tunisian individuals. ^138^ The next highest-ranked *k*-mer impacted 1-Acylglycerol-3-Phosphate O-Acyltransferase 4 ( *AGPAT4*), which has been proposed as required for triple-negative breast cancer progression. ^139^ This ranked *k*-mer list and genes impacted provide a resource to “fish” for genes relevant to breast cancer metastasis.

### Imavirus

When deriving putative viral integration sites from RNA-seq, sites may be more likely to be detected if coming from highly expressed loci. The Tg26 mouse reanalyzed in the present study was made from pronuclear injection of pNL4–3 restriction products. ^61^ Such insertion depends entirely on host DNA repair machinery on nuclear DNA with nuclear topology distinct from human cells which HIV more easily infects. As such, many of the sites missed during the RNA-seq interrogation may have been missed due to low levels of expression at those loci, or those parts of the complex insertion site. Many viruses have the capability of integrating into host genomes, leading to DNA damage and gene disruption. Accurately identifying virus integration sites and potentially disrupted genes is important to fully understand their impact on disease severity. However, identifying virus integration sites from genomic DNA is challenging and there are not many bioinformatics tools available to reliably detect viral presence or integration events. Here, we developed Imavirus, a bioinformatics approach that identifies putative virus integration sites (pIS) in public data. Using unbiased RNA-seq datasets, Imavirus aimed to identify pIS and to pinpoint clinically relevant viral integration sites, especially those that may affect cell function and possibly contributing to disease and/or antiviral responses and possibly contributing to virus fitness. Imavirus is a community resource that aids researchers by enhancing our knowledge of viral infection and improving disease severity prediction of viral infections. During the hackathon we were able to verify a previously reported integration site on mouse chr8 which can be seen in our GitHub repository cited in the Software Availability section.

Future work should explore the datasets we scoped out in SRA for more physiological systems such as animal models or stable cell lines to identify more putative insertion sites. Another important limitation of the positive control set explored here is that the Tg26 mouse has approximately 15 copies of HIV integrated at the same loci. ^125^^,^^126^ While HIV signal may be coming from multiple loci, when considering junctions, most of the signal seemed to be coming from an HIV copy with run-on transcripts in chr8. Natural human infections would have distinct insertion sites, making them harder to spot with these approaches.

### RPG

The human reference genome has served as a foundation for human genetics and genomics studies. Despite its countless applications, the current human reference genome assembly (GRCh38) harbors several gaps and missing nucleotides (‘N’ characters) that hinder comprehensive analysis. Therefore, complete T2T genome references are essential to make sure all the genomic variants are discovered and analyzed. Here, we implemented a pipeline that incorporates 1000 Genome Project common alleles and avoids stop codons into the T2T-CHM13 genome sequence, in order to provide a complete human reference sequence for diverse sequencing data analyses.

## Conclusions

The results of the 2021 Baylor College of Medicine/DNAnexus hackathon described here represent novel work that pushes the field forward for human, plant, and viral genome SV detection. All are needed to further current findings about diversity and the complexity of organisms and their genotypes. To further facilitate this progress in a FAIR-compliant manner, 59 people, across the world with different professional backgrounds, came together in October 2021 to complete or further eight groundbreaking prototypes.

## Next steps

Some directions that we think will be impactful in the future are:

*k*-mer analyses avoid reference and mapping biases through a reference-free approach. We endeavor to create tools for *k*-mer analysis which work with both short- and long- read sequencing technologies. An example use case is the identification of rare variants can help in diagnostic purposes to identify disease biomarkers and therapeutic targets for personalized medicine. Along similar lines, crop breeding research can benefit from identifying markers associated with disease resistance.

Specifically, we would improve the kTom tool to enable quantification of *k*-mers to detect potential copy-number changes, and this would be particularly relevant for disease resistance loci, which often contain gene copy-number variations. ^140^

To achieve these outcomes, additional modules can be added to the kTom code base. For example, for disease-resistance introgression that is known to be in some but not all of samples in a collection, the *k*-mer frequency matrix can be filtered to keep low-middle frequency *k*-mers. With or without this filtering step, a distance matrix can be computed from the *k-*mer frequency matrix and used for hierarchical clustering to suggest sets *k*-mers introgressed together. The resulting output can then be used for validation complemented by curated and known loci, and later for phenotypic association.

For clearer virus integration site mapping, long read DNA sequencing is preferable to short reads, but these types of data are sparse in major public repositories. The present hackathon scoped out sequences from the Sequence Read Archive and subset viruses and controls relevant for integration studies. Therefore, future work is needed to compare short-read datasets to long-read generated stable vs transient insertion sites in order to improve our understanding of the effects on viral replication, host gene regulation, and disease.

To inform clinical significance of SVs for clinicians and researchers, GeneVar2 is a comprehensive tool for understanding the impact of SVs on disease. To expand users’ ability to identify and communicate key SV findings, Samplot, ^141^ a multi-sample structural variant visualizer, will be integrated with GeneVar2. Subsequent development will focus on further cloud integration and new output options, such as research reports and the ability to use the application off-line.

The annual nature of this hackathon has seeded teams and projects that are often ongoing for multiple years, resulting in mature software products. Other annual hackathons, particularly the NBDC/DBCLS (Japan) and ELIXIR (Europe) bio-hackathons, have seen the same. In this vein, we expect to see many of the projects that have been seeded here continue next year, and possibly in other hackathons.

## Data availability

### Underlying data

The data used for these projects were obtained from publicly accessible repositories and are available in Table 1.

**Table 1.** Lists the data source utilized by each tool developed during the hackathon.

Tool name	Data source utilized
STRdust	GRCh38 human reference genome: https://ftp.ncbi.nlm.nih.gov/genomes/all/GCF/000/001/405/GCF_000001405.39_GRCh38.p13/GCF_000001405.39_GRCh38.p13_genomic.fna.gz SL4.0 tomato genome: https://solgenomics.net/ftp//tomato_genome/assembly/build_4.00/S_lycopersicum_chromosomes.4.00.fa.gz
kTom	100 Tomato Consortium: whole genome data of 84 tomatoes, BioProject PRJEB5235 - https://www.ncbi.nlm.nih.gov/bioproject/236988
INseption	GIAB HiFi data set (fastq files): https://ftp-trace.ncbi.nlm.nih.gov/ReferenceSamples/giab/data/AshkenazimTrio/HG002_NA24385_son/PacBio_CCS_15kb/
GeneVar2	Gene association with phenotype disorders in OMIM: https://maayanlab.cloud/static/hdfs/harmonizome/data/omim/gene_list_terms.txt.gz R clusterProfiler annotation for Disease Ontology, DisGeNET, Network of Cancer Gene, Gene Ontology, KEGG pathway: https://guangchuangyu.github.io/software/clusterProfiler/ dbVar, known clinical SV annotation, GRCh38: http://ftp.ncbi.nlm.nih.gov/pub/dbVar/data/Homo_sapiens/by_study/tsv/nstd102.GRCh38.variant_call.tsv.gz GENCODE v35 gene annotation: http://ftp.ebi.ac.uk/pub/databases/gencode/Gencode_human/release_35/gencode.v35.annotation.gff3.gz gnomAD pLI information: https://azureopendatastorage.blob.core.windows.net/gnomad/release/2.1.1/constraint/gnomad.v2.1.1.lof_metrics.by_gene.txt.bgz gnomAD-SV BED file with allele frequencies: https://datasetgnomad.blob.core.windows.net/dataset/papers/2019-sv/gnomad_v2.1_sv.sites.bed.gz
K-var	Whole exome sequencing data of NCI-60 dataset, BioProject PRJNA523380. Breast cancer accession numbers: SRR8619035, SRR8619036, SRR8619038, SRR8619044, SRR8619110, SRR8619113, SRR8619154, SRR8619076, SRR8619133, SRR8619134, SRR8618981, SRR8619186 GRCh38 human reference genome: http://ftp.ebi.ac.uk/pub/databases/gencode/Gencode_human/release_38/GRCh38.primary_assembly.genome.fa.gz GRCh38 Gencode gene annotations: http://ftp.ebi.ac.uk/pub/databases/gencode/Gencode_human/release_38/gencode.v38.primary_assembly.annotation.gff3.gz
Imavirus	SRA RNA-seq test data: SRR10302267 *.* The accession for the pNL4–3 used to make the Tg26 mouse is GenBank: AF324493.2, and this was used to explore data in IGV. The mm10 mouse genome was used to visualize cognate integration site(s) on mouse chr8. Accession lists in GitHub repository listed in the software availability section.
RPG	Complete genome of CHM13 T2T v2.0, BioProject PRJNA559484: https://www.ncbi.nlm.nih.gov/assembly/GCA_009914755.4

## Software availability

### STRdust

Source code available from: https://github.com/collaborativebioinformatics/STRdust

Release version: 0.2.

Archived source code at time of publication: https://doi.org/10.5281/zenodo.6467829. ^142^

License: MIT.

### kTom

Source code available from: https://github.com/collaborativebioinformatics/kTom

Release version: 0.2.

Archived source code at time of publication: https://doi.org/10.5281/zenodo.6467823. ^143^

License: MIT.

### INSeption

Source code available from: https://github.com/collaborativebioinformatics/InSeption

Release version: 0.2.

Archived source code at time of publication: https://doi.org/10.5281/zenodo.6467818. ^144^

License: MIT.

### GeneVar2

Source code available from: https://github.com/collaborativebioinformatics/GeneVar2

Release version: 0.2.

Archived source code at time of publication: https://doi.org/10.5281/zenodo.6467837. ^145^

License: MIT.

### Cov2db

Source code available from: https://github.com/collaborativebioinformatics/cov2db

Release version: 0.2.

Archived source code at time of publication: https://doi.org/10.5281/zenodo.6467825. ^146^

License: MIT.

### K-var

Source code available from: https://github.com/collaborativebioinformatics/kvar

Release version: 0.2.

Archived source code at time of publication: https://doi.org/10.5281/zenodo.6467850. ^147^

License: MIT.

### Imavirus

Source code available from: https://github.com/collaborativebioinformatics/imavirus

Release version: 0.2.

Archived source code at time of publication: https://doi.org/10.5281/zenodo.6467774. ^148^

License: MIT.

### RPG

Source code available from: https://github.com/collaborativebioinformatics/RPG_Pikachu

Release version: 0.2.

Archived source code at time of publication: https://doi.org/10.5281/zenodo.6467816. ^149^

License: MIT.
