# Supplementary material for: The third international hackathon for applying insights into large-scale genomic composition to use cases in a wide range of organisms
Source: F1000Res. 2022 May 16;11:530. [Version 1] doi: 10.12688/f1000research.110194.1 (PMC9557141; doi:10.12688/f1000research.110194.1)
Supplement: Peer review history

# Peer review: Reviewer response for version 1

**DOI:** 10.5256/f1000research.121770.r149877

**Published:** 2022-10-19

**Authors:** Quan Long

**Affiliations:** 1 University of Calgary, Calgary, Canada

In the manuscript, the authors have reported “The third hackathon for applying insights into large-scale genomic composition to use cases in a wide range of organisms” that happened in Baylor College of Medicine in 2021.

 Detailed descriptions of eight software packages focusing on structural variation detection have been provided. The data for benchmarking are also mentioned. However, I do not see any quantitative results regarding to the evaluation of the software. For instances: what are the true/false positives for the tools? What are the advantages/disadvantages of different tools when comparing to each other? The manuscript focuses more on describing the algorithms and the data, which are important. However, without an evaluation, this seems to be an unfinished work. If the evaluation could be added, this looks an excellent review and comparison of SV tools.

 Also it is clear that different authors have written their pieces and then submitted. The work looks quite segmented without smoothing.

Are the conclusions about the tool and its performance adequately supported by the findings presented in the article?

No

Is the rationale for developing the new software tool clearly explained?

Partly

Is the description of the software tool technically sound?

Partly

Are sufficient details of the code, methods and analysis (if applicable) provided to allow replication of the software development and its use by others?

Yes

Is sufficient information provided to allow interpretation of the expected output datasets and any results generated using the tool?

No

Reviewer Expertise:

Genomics, Genetics, Machine Learning

I confirm that I have read this submission and believe that I have an appropriate level of expertise to confirm that it is of an acceptable scientific standard, however I have significant reservations, as outlined above.

# Peer review: Reviewer response for version 1

**DOI:** 10.5256/f1000research.121770.r149876

**Published:** 2022-10-12

**Authors:** Pedro G. Ferreira

**Affiliations:** 1 University of Porto, Porto, Portugal

Kimberly Walker and colleagues presents a manuscript that reports the results of a workshop meeting where different groups have applied their developed tools for the analysis of genomic variation.

 The manuscript is written in an unconventional way, which is hard to follow.

 My main concern with the manuscript is that I did not find a conducting line or a specific goal, rather than reporting the methods that were applied. The title mentions a wide range of organisms. In the introduction, it is mentioned a focus on tomatoes, plants, Sars-cov2, and some methods like INSeption analyse human data from Genome in a Bottle project. Overall, that is too confusing.

 The text focuses on describing the tools and their workflows. It would be interesting to discuss the results from a qualitative and quantitative point-of-view. This way, one would obtain a better intuition of the relevance and utility of the tool.

 What are the results of applying these tools in tomato genome? It would be very interesting to summarize the novel findings on the tomato genome or the human genome from the different views of applying all these tools. For instance, in the INSeption section it is mentioned allele frequency analysis. I’m very curious about these results, but these were not discussed at all.

 From the quantitative point of view, what are the computational demands of these tools? What are the performance gains of using a k-mer approach when compared with other approaches?

 Overall, without a clear focus on the goals and the reporting of the insights gained, the manuscript is of little use. Therefore, I recommend a substantial re-organization and improvement of the manuscript.

Are the conclusions about the tool and its performance adequately supported by the findings presented in the article?

No

Is the rationale for developing the new software tool clearly explained?

No

Is the description of the software tool technically sound?

Partly

Are sufficient details of the code, methods and analysis (if applicable) provided to allow replication of the software development and its use by others?

Yes

Is sufficient information provided to allow interpretation of the expected output datasets and any results generated using the tool?

No

Reviewer Expertise:

Transcriptomics

I confirm that I have read this submission and believe that I have an appropriate level of expertise to confirm that it is of an acceptable scientific standard, however I have significant reservations, as outlined above.

# Peer review: Reviewer response for version 1

**DOI:** 10.5256/f1000research.121770.r149878

**Published:** 2022-09-23

**Authors:** Nguyen Quoc Khanh Le

**Affiliations:** 1 Taipei Medical University, Taipei, Taiwan

In this study, the authors briefly went through all algorithms in the third international hackathon for applying insights into large-scale genomics composition. Overall, it is well-written and holds potential for indexing. I just have some minor comments as follows:

1. I'm wondering whether the authors included all projects from the hackathon or not. Any reasons to exclude the other projects out of this analysis?
2. Adding more use cases to each algorithm would be more interesting.
3. The authors should add more discussions to show the biological insights of each tool/algorithm.

Are the conclusions about the tool and its performance adequately supported by the findings presented in the article?

Yes

Is the rationale for developing the new software tool clearly explained?

Yes

Is the description of the software tool technically sound?

Yes

Are sufficient details of the code, methods and analysis (if applicable) provided to allow replication of the software development and its use by others?

Yes

Is sufficient information provided to allow interpretation of the expected output datasets and any results generated using the tool?

Yes

Reviewer Expertise:

Genomics analysis

I confirm that I have read this submission and believe that I have an appropriate level of expertise to confirm that it is of an acceptable scientific standard.

# Peer review: Reviewer response for version 1

**DOI:** 10.5256/f1000research.121770.r139299

**Published:** 2022-06-22

**Authors:** Rodolfo Aramayo

**Affiliations:** 1 Department of Biology, Texas A&M University, College Station, TX, USA

**Introduction:**

 In this manuscript, Kimberly Walker and colleagues describe and summarize the results of *"The third hackathon for applying insights into large-scale genomic composition to use cases in a wide range of organisms."*

 This virtual hackathon took place in October 2021 and encompassed a total of 59 scientists from 14 countries and 13 U.S. states.

 In this manuscript, the authors describe the evaluation of eight software packages aimed at identifying Structural Variants (SVs) by using mainly k-mers-based tools, among others.

 This manuscript addresses key problems in Computational Genomics. Importantly, unlike before, the 2021 hackathon features work on plants, which provides an important alternative point of view to the human-focused research of previous hackathons.

 Below you will find a series of general and specific recommendations that will, in my opinion, significantly improve the readability and overall quality of this work, while correcting a series of issues I have found associated with both the online version and the downloaded PDF version of the manuscript.

**General Comments:**

 I hope you will agree with me that this is, by no means, a "standard" manuscript. Yet, the manuscript was written trying to adjust the logic of the text to that of a "standard" manuscript. It is very hard to follow the logic of what was described in the text, as the text is presented.

 Let me explain: any potential reader that is trying to learn, understand and follow what was done with any one of the software packages here described would have an extremely hard time trying follow the logic of a given tool under the current manuscript format.

 The current format is fragmented. Information related to a given software package is scattered around the text. It is hard to follow.

 This is why I strongly suggest to reorganize the text of the manuscript as follows:

 1. Abstract

 2. Introduction to the general topic **only** (not to the different packages used)

 3. Methods. Describe the methods general to all the work (Cloud Computing)

 4. STRdust: Detect and genotype short tandem repeats

 4.1. Introduction to STRdust

 4.2. Experimental Rationale, Methods and Discussion for STRdust

 4.3. Next Steps or Recommended Future Directions for STRdust

 5. kTom: k-mers for profiling tomato introgressions

 5.1. Introduction to kTom

 5.2. Experimental Rationale, Methods and Discussion for kTom

 5.3. Next Steps or Recommended Future Directions for kTom

 6. cov2db

 6.1. Introduction to cov2db

 6.2. Experimental Rationale, Methods and Discussion for cov2db

 6.3. Next Steps or Recommended Future Directions for cov2db

 ...

 ...

 ...

 X. Conclusions

 Y. Tables

 Y.1. Table 1. Lists the data source utilized by each tool developed

 during the hackathon.

 Y.2 Table 2. A table compiling the Data, and Software Availability and

 others.

 V. Acknowledgements

 Z. References

 Regarding what I call in the example above section Y.2, I would like to see the data associated Data, Software availability, and others organized as a single table with the following format or something similar:

 =========================

 Table 2. Software availability

                   Row 1                 Row 2

 Column 1: Tool Name           STRdust

 Column 2: Source code        https://github.com/collaborativebioinformatics/STRdust 

 Column 3: Version No.          0.2

 Column 4: Link to Archive    https://doi.org/10.5281/zenodo.6467829 

 Column 5: License                MIT

 =========================

 In summary, I think that the format described will be easier to read, as it will contain all the information and figures relating to a piece of software together. It will follow a logical flow. As far as I am concerned, this is a better way to present this work. It is not a "conventional" way, but then again, this is not a "conventional" manuscript.

**Specific Comments:**

**Comment 01:**

 The paragraph:

*"These genomic variants are typically classified as deletions, duplications, insertions, inversions, and translocations describing different combinations of DNA gains, losses, or rearrangements. Copy number variations (CNVs) are a particular subtype of SVs mainly represented by deletions and duplications. SVs are typically described as single events, although more complex scenarios involving combinations of SV types exist.1,2"*

 Should read:

 These genomic variants are typically classified as deletions, duplications, insertions, inversions, and translocations describing different combinations of DNA losses, gains and/or rearrangements. SVs are typically described as single events, although more complex scenarios involving combinations of SV types exist. Copy number variations (CNVs) are a particular subtype of SVs mainly represented by deletions and duplications. 1,2

 Rationale: As originally written, the paragraph starts describing Structural Variants (SVs), then switches to Copy Number Variations (CNVs), and the returns to SVs. I would like to see the ideas of SVs presented together.

**Comment 02:**

 The paragraph:

*"In October 2021, 59 researchers from 14 countries participated virtually in the third Baylor College of Medicine & DNAnexus hackathon, focusing on interrelated topics such as SVs, short tandem repeats (STRs), k-mer profiling, viruses, reference refinement and annotation."*

 Should read:

 In October 2021, 59 researchers from 14 countries participated virtually in the third Baylor College of Medicine & DNAnexus hackathon, focusing on interrelated topics such as k-mer profiling, short tandem repeats (STRs), SVs, reference refinement, annotation and viruses.

 Rationale: I would like to see the presented topics sorted according to those that are related to large genomes and then small genomes (i.e., viruses). This order should also be applied to the rest of the manuscript.

**Comment 03:**

 The paragraph:

*"The international hackathon focused on nine softwares to answer these questions; eight of which we present in this paper: STRdust, kTom,INSeption, GeneVar2, cov2db, K-var, Imavirus, and a Reference Panel Generator (RPG) for diverse sequencing data analysis. Several emergent themes became apparent over the course of the hackathon."*

 Should read:

 The international hackathon focused on nine software packages (eight of which we present in this paper), to answer these questions: K-var, kTom, STRdust, INSeption, GeneVar2, cov2db, RPG, and Imavirus. Several emergent themes became apparent over the course of the hackathon.

 Rationale: I think it reads better and lists the virus-related package last (see Comment 02).

**Comment 04:**

 The paragraph:

*"Nucleotide sequence substrings of length k (k-mers) continue to prove useful in SV work and in genomics, however, the time needed to assess the frequency of SVs presents a resource problem.7 The reduction of the computational resources required to complete an SV assessment in a genome would allow greater amounts of SV data to be processed in genomic workflows. Many bioinformatic tools currently used to locate genomic SVs use a sliding window alignment technique, which can be time-consuming.8,9 However, implementing a k-mer based approach to create a pool of reference k-mers of known SVs, the annotation speed of variation in new genomes might be increased.10,11 k-mers have also been used in alignment-free methods, bypassing the need for reference genomes.12"*

 Needs serious rearrangement. I read this paragraph as follows:

 Here you are telling me that k-mers are useful for SV work:

*"Nucleotide sequence substrings of length k (k-mers) continue to prove useful in SV work and in genomics,"*

 Here you are telling me that generating SV is computationally expensive:

*"however, the time needed to assess the frequency of SVs presents a resource problem.7"*

 Here you are telling me that if we were to reduce the time it takes to compute them, they would then be more likely to be incorporated in genomic workflows:

*"The reduction of the computational resources required to complete an SV assessment in a genome would allow greater amounts of SV data to be processed in genomic workflows."*

 Here you are describing that time consuming sliding windows alignment techniques are currently used to locate genomic SVs:

*"Many bioinformatic tools currently used to locate genomic SVs use a sliding window alignment technique, which can be time-consuming.8,9"*

 Here you are telling me that if we were to implement a k-mer-based approach, the annotation speed would increase:

*"However, implementing a k-mer based approach to create a pool of reference k-mers of known SVs, the annotation speed of variation in new genomes might be increased.10,11"*

 Here you are stating that k-mer have been used in alignment-free methods:

*"k-mers have also been used in alignment-free methods, bypassing the need for reference genomes.12"*

 I would like to see these ideas presented as follows (re-do the text):

 First, introduce k-mers and how k-mers are being used in genomics:

*"k-mers have also been used in alignment-free methods, bypassing the need for reference genomes.12"*

 Second, state that k-mers are useful:

*"Nucleotide sequence substrings of length k (k-mers) continue to prove useful in SV work and in genomics,"*

 State that the way SV are calculated is time-consuming:

*"Many bioinformatic tools currently used to locate genomic SVs use a sliding window alignment technique, which can be time-consuming.8,9"*

 Third, state that calculating SV is expensive:

*"however, the time needed to assess the frequency of SVs presents a resource problem.7"*

 Fourth, tell us why using k-mers might be better:

*"However, implementing a k-mer based approach to create a pool of reference k-mers of known SVs, the annotation speed of variation in new genomes might be increased.10,11"*

 Finally, conclude how faster calculation is likely to result on SV data to be readily incorporated in existing genomic workflows, thus improving the overall genome annotation:

*"The reduction of the computational resources required to complete an SV assessment in a genome would allow greater amounts of SV data to be processed in genomic workflows."*

**STRdust Specific Comments:**

**Comment 05:**

 In following Resource IDs:

 (RRID:SCR_010233)

 (RRID:SCR_017990)

 (RRID:SCR_003756)

 (RRID:SCR_002796)

 (RRID:SCR_018801)

 their links do not work because the RESEARCH RESOURCE IDENTIFICATION PORTAL has changed. The only way I have found to access the resource is by accessing the legacy RRID website ( https://scicrunch.org/resources-legacy/).

 Regarding resource SCR_002796, it points to a github repository ( https://github.com/alibashir/pacmonstr), which, in my opinion is the link it should be used (added as a reference?). The same can be said about resource SCR_018801, that points to the https://github.com/davidebolo1993/TRiCoLOR, repository.

 In general, I think that GitHub repositories that have not been archived via databases like Zenodo, should be linked directly to GitHub.

**Comment 06:**

 In the following paragraph:

*"To mitigate this issue, several long-read STR calling methods have been developed in recent years, including PacmonSTR21(RRID:SCR_002796), NanoSatellite,22 TRiCoLOR23(RRID:SCR_018801), and Straglr24 -- however, their usability remains limited due to platform and/or computational demands"*

 You state that either the usability or computational demands of the packages: PacmonSTR, NanoSatellite, TRiCoLOR, and Straglr, are problematic.

 In fairness to the authors of those packages, you should provide data supporting that statement.

 In the legend of Figure 1 you clearly state:

*"We evaluated STRdust by comparing the results of simulated STR expansions produced by SimiSTR based on the human (Genome Reference Consortium Human Build 38, GRCh38) and tomato (Solanum lycopersicum 4.0, SL4.0) reference genomes, to two novel tools: Straglr24 and TRiCoLOR.23"*

 And in the text, you state:

*"STRdust results were compared to TRiCoLOR 1.1,23 and Straglr 1.1.124 using default parameters."*

 But you never really showed any data that would allow an independent observer to reach the same conclusion you claim to have reached.

 In my opinion, this is not fair to the developers of the other packages and should be corrected.

**kTom Specific Comments:**

**Comment 07:**

 Please define: *"k-mers with low-mid range frequencies"*

**Comment 08:**

 Please state the length or range of lengths of the Illumina reads used in these experiments.

 It is not clear to me how the read length affects the outcome of this analysis (has anyone tested this parameter?).

**Comment 09:**

 Also, it is important to state the k-mer length or k-mer length range that was used for this analysis.

 What is the minimum k-mer length that is calculated/used by this package?

**Comment 10:**

 As I suspect that the read coverage will have a profound effect on these experiments, therefore, I would like to see a table (or Supplementary table), where the coverage for the different datasets analyzed is presented.

**Comment 11:**

 Where the FastQ read lengths normalized in these experiments?

 If they were not, then it is hard for me to evaluate the meaning of the results displayed in Figure 2. Again, data requested in Comment 09 is important.

**Comment 12:**

 The link to resource RRID:SCR_014583, is not working. Also, this resource should link directly to the FastQC web site ( https://www.bioinformatics.babraham.ac.uk/projects/fastqc/).

**Comment 13:**

 Similarly, link to resources SCR_013001, SCR_005491, SCR_017014, and SCR_017270 are not working.

**Comment 14:**

 When searched manually, resource SCR_013001 links to the Sourceforge link: https://sourceforge.net/projects/flexbar/, but in there the Flexbar version 1.4.0, used in the work is not present. The oldest version present there is for version 2.2. Also, the official Flexbar website has been moved to GitHub ( https://github.com/seqan/flexbar). Version 1.4.0, is not available there either, thus a person wanting to reproduce these results using the exact same versions presented in this manuscript, would be unable to do so.

 This needs to be corrected.

**Comment 15:**

 Resources SCR_005491 and SCR_017014, should link directly to GitHub ( https://github.com/gmarcais/Jellyfish and https://github.com/schatzlab/genomescope, respectively), and resources SCR_017270 and SCR_019186, should link directly to Bioconductor ( https://bioconductor.org/packages/release/bioc/html/ComplexHeatmap.html) and CRAN ( https://cran.r-project.org/web/packages/tidyverse/index.html), respectively.

**Comment 15:**

 The resolution of Figure 2 must be increased. When the PDF file is displayed on a large screen the figure is extremely pixelated and hard to interpret.

**INseption Specific Comments:**

**Comment 16:**

 The resources for

 SCR_010910 - BWA,

 SCR_018550 - Minimap2,

 SCR_017620 - NGMLR,

 SCR_004603 - DELLY,

***SCR_004603 - SNIFFLES \<==Note wrong ID!,***

 SCR_015880 - Canu,

 SCR_017016 - Flye,

 SCR_005227 - BCFTools,

 SCR_002105 - SamTools,

 SCR_008394 - Python, and

 SCR_015880 - Canu

 Have links not working and, again, in my opinion should point to their GitHub or SourceForge repositories.

 I really appreciate the work of trying to make uniform the web addresses citations, but in the long term, as the repositories get updated, it will create a problem.

 Please consider adding these links as part of the references.

 Modern Reference Managers like Paperpille ( https://paperpile.com/app), do an excellent job doing this.

**Comment 17:**

 You state that: *"We filtered out SVs that were supported by less than 10 reads using bcftools 1.12"*

 Bcftools is a complex set of scripts. Please provide the exact command used.

**Comment 18:**

 Figure 3 is very hard to understand. The problem is that you are using lower-case letters and upper-case letters to refer to the different panels. Do not do that. Use Panel A, SubPanel I, etc.

 This figure needs to be edited or replaced and the figure legend needs to be re-written so as to make it digestible. It is not clear what are you trying to show: are you showing haplotypes? Are you showing reference and experimental regions? Why is the F panel on the right? And the C, D, and E panels on the left? This is confusing. I understand you are trying to save space, but the logic must be presented sequentially.

**Comment 19:**

 In the section: *"Clustering unmapped reads"*

 You state:

*"To be able to assemble a sequence from all unmapped reads, we tried several approaches. We attempted to identify clusters of reads using the LROD version 1.094 package, which we found unsuitable for our purposes due to long runtimes. More successfully, we used the program CARNAC-LR version 1.0.095 to build clusters of reads using Minimap2 version 2.22 aligner32 and a subsequent k-mer based clustering approach."*

 Here, you need to define what you mean by *"due to long runtime"*.

 Also, you need to define and describe what you mean by:

*"and a subsequent k-mer based clustering approach."*

 You need a reference to a publication or to a script name or to a repository.

**Comment 20:**

 In the section: *"Identifying integration sites for assembled clusters"*

 You state:

*"Having successfully assembled contigs for N = 15 read clusters using Canu v2.236(RRID:SCR_015880), we searched for overlap of these contigs with the breakpoint regions of 30 previously identified long insertion sites."*

 How were those 30 regions previously identified?

**Comment 21:**

 Also related to Comment 20, are you stating that you re-identified regions that were previously identified as containing insertions?

 If this is the case, can your experimental logic be applied to identify unknown insertion points?

 If the answer is yes, then why have you not done that?

 Can you expand Figure 3 to clarify this point?

**Comment 22:**

 Please comment on the Genome Coverage needed for INseption to work as expected.

 Has anyone tested the effects of Genome Coverage on INseption's performance?

**Comment 23:**

 The most important part that must be addressed in this section is the fact that while you have explained the logic of using sample reads to detect insertions in the reference genome, by definition, in comparing genomes from the same population, an insertion in Genome A is a deletion in Genome B and an insertion in Genome B, is a deletion in Genome A. So, it is not clear to me how your experimental logic would be applied to a deletion present in the reference genome and an insertion in the sample reads. As things are explained here, it looks to me that you seem to have only solved 50% of the problem.

**GeneVar2 Specific Comments:**

 Comment 16 applies to this section.

**Comment 24:**

 How is GeneVar2 different or better or what are its advantages when compared to seeing this same information in a good old genome browser like Ensembl? Please elaborate.

**Comment 25:**

 Also, I think it would really help if you could add a figure showing the how a gene like BRCA2 (ENSG00000139618), displays in GeneVar2. That would help people not familiar with the **GeneVar2** browser to be able to see it in action.

**cov2db Specific Comments:**

 Comment 16 applies to this section.

**Comment 26:**

 In the statement:

*"Minimal system requirements for a local cov2db instance are dictated by the mongoDB requirements with the key limiting factor being RAM used. Large variant databases will consume substantial amounts of RAM, and we suggest hosting those on dedicated high memory compute servers."*

 You need to be more specific. Define *large database,* and be more specific about RAM usage. Also, when recommending to use dedicated high memory compute servers, please be more specific on the minimal requirements needed.

**Comment 27:**

 In the statement:

*"Our current design supports input VCFs generated by LoFreq117 (RRID:SCR_013054) or converted into VCFs from the iVar118 output via provided script."*

 Please give the name of the *"provided script".*

**Comment 28:**

 To truly appreciate the package cov2db in action, I would recommend the authors present a screenshot of an actual data display of a region of the virus in the R Shiny app.

**K-var Specific Comments:**

 Comment 16 applies to this section.

**Comment 29:**

 The URL ( http://www.dtp.nci.nih.gov/) present in the resource RRID:SCR_003057 (when searched manually, because the link does not work...) point to a dead web site.

**Comment 30:**

 The statement:

*"k-mer frequencies were obtained for each sample, using the tool Jellyfish version 2.3.0."*

 Needs clarification. What kind of data was used to obtain the k-mers? FastQ files? BAM files? Tables?

**Comment 31:**

 You mention that you used *"whole exome sequencing of the NCI-60 dataset"*, but based on the data you gave me I was unable to find that dataset. Even when I searched the NCBI-SRA website using the terms *"NCI-60 cancer"* I was not able to easily identify the exome datasets in question.

 Please give specific links to the data you used in these experiments.

**Comment 32:**

 It is totally unclear to me how the data coming from these different samples compare to each other in terms of coverage/number of reads.

 It would help if you were to provide a table with this data. K-mer frequency is dependent on coverage.

**Comment 33:**

 Please provide the name of the *"custom script"* used to tabulate the data.

 Is this script part of your submission?

**Comment 34:**

 Please define *"low frequency k-mers"*

**Comment 35:**

 How were the control and test datasets pre-defined? And how you implemented the TF-IDF test? (R Script?, Python Script?).

**Imavirus Specific Comments:**

 Comment 16 applies to this section.

**Comment 36:**

 The statement:

*"During the hackathon we were able to verify a previously reported integration site on mouse chr8 which can be seen in our GitHub repository cited in the Software Availability section."*

 How did you do this? What commands did you use? Where is your computational pipeline?

**Comment 37:**

 The statement:

*"Using unbiased RNA-seq datasets, Imavirus aimed to identify pIS..."*

 Please define *"unbiased"*

**Comment 38:**

 The statement:

*"Future work should explore the datasets we scoped out in SRA for more physiological systems such as animal models or stable cell lines to identify more putative insertion sites."*

 What do you mean by: *"more physiological systems such as animal models or stable cell lines to identify more putative insertion sites"*

 This statement makes no sense to me. A bacterial cell does not have less Physiology than a Human Liver cell. It has a *different* physiology. Please rephrase this idea.

**Comment 39:**

 Based on what was presented in this manuscript Imavirus is an idea, not a tool. The authors are not presenting a logical code-based pipeline to complete this analysis.

 The Zenodo repository contains tables and figures, not code, and although this is a nice idea, I cannot understand why this was included on this manuscript. The tool Imavirus as presented is an idea and should not be part of this report. Alternatively, the authors should provide a viable computational pipeline that can be used to perform a similar analysis. I was unable to find not a single draft text page containing a list of commands.

 As this *"pipeline"* is presented is completely unreproducible and useless.

 This section is by far below the standards of the other tools presented. It should either be removed or re-done. As it is, it hurts the manuscript as it does not follow the F1000 Condition for publication:

*"Are sufficient details of the code, methods and analysis (if applicable) provided to allow replication of the software development and its use by others?"*

 In addition, the authors make no mention of competing tools like ViFi, VirTect, and VIRUSBreakend.

 In fact, when they mention:

*"However, identifying virus integration sites from genomic DNA is challenging and there are not many bioinformatics tools available to reliably detect viral presence or integration events."*

 Without mentioning competing tools, is not fair to the authors of those other competing tools and, in my opinion, it is misleading.

 I have to conclude that, after reading this section I am left with the taste that Imavirus is an idea that is being presented so as to be "sold" to be incorporated into a commercial product.

**RPG Specific Comments:**

 Comment 16 applies to this section.

**Comment 40:**

 In the legend of Figure 8 you state:

*"Only common alleles (\>5% allele frequency (AF)) in the variant call set are retained."*

 and

*"Subsequently, common allele calls are replaced with CHM13 rare alleles in CHM13 FASTA genome sequence."*

 Please elaborate how you did that.

 Also elaborate if you do that for both haplotypes or not.

**Comment 41:**

 It would help to have a table with the actual numbers of variants calls, non-pathogenic variants, pathogenic variants, number of in-frame stop codons, etc.

**Comment 42:**

 It is not clear to me the origin of the list of 1KGP common alleles. Where those downloaded or calculated? And, if they were calculated, can you provide their list?

**Conclusion:**

 This is a good piece of work. Here, I am offering a series of suggestions that, I am sure, will improve the overall quality of this work.

Are the conclusions about the tool and its performance adequately supported by the findings presented in the article?

Partly

Is the rationale for developing the new software tool clearly explained?

Yes

Is the description of the software tool technically sound?

Yes

Are sufficient details of the code, methods and analysis (if applicable) provided to allow replication of the software development and its use by others?

No

Is sufficient information provided to allow interpretation of the expected output datasets and any results generated using the tool?

Partly

Reviewer Expertise:

Genetics, Genomics, Computational Genomics, Epigenetics, Fungal Genetics and Biology, Metazoan Genome Organization and Digital Biology

I confirm that I have read this submission and believe that I have an appropriate level of expertise to confirm that it is of an acceptable scientific standard, however I have significant reservations, as outlined above.
